# Dynamics-enhanced molecular property prediction guided by deep learning

**DOI:** 10.1371/journal.pcbi.1014515

**Published:** 2026-07-16

**Authors:** Qiang Liu, Debby Dan Wang, Weiqing Guo, Yuting Huang, Xizhao Wang

**Affiliations:** 1 College of Computer Science and Software Engineering, Shenzhen University, Shenzhen, Guangdong, China; 2 School of Science and Technology, Hong Kong Metropolitan University, Ho Man Tin, Kowloon, Hong Kong; 3 The Guangdong Key Laboratory of Intelligent Information Processing, Shenzhen University, Shenzhen, Guangdong, China; University of California Riverside, UNITED STATES OF AMERICA

## Abstract

Molecular property prediction (MPP) is a key challenge in computational biology and drug discovery. Traditional approaches mostly rely on feature representations yielded from static structures of molecules, ignoring their dynamic nature. The scarcity of dynamics data in public databases and the complexity of learning such high-dimensional data made it more difficult for dynamics-involved studies. Accordingly, we built a series of dynamics datasets for MPP tasks by performing comprehensive molecular dynamics (MD) simulations on different molecules. In addition, we proposed a dynamically enhanced molecular representation (DEMR) method with multiple sampling strategies for the dynamics frames. Besides, two deep learning pipelines were employed for mapping DEMR to the molecular properties in various tasks. Our models achieved better performance in different MPP tasks, with practical guidance in efficient frame selection. This study highlights the significance of integrating MD data into MPP tasks and opens new avenues for structure-based drug design. The generated MD datasets are publicly available in a *Zenodo* repository at https://doi.org/10.5281/zenodo.15788151, and the code is available in a *GitHub* repository at https://github.com/liuqiang-blib/MPP-using-DEMR.git.

## Author summary

Predicting molecular properties is a central problem in computational biology, drug discovery and chemical safety evaluation. Molecules are dynamic objects in solution and constantly undergo conformational changes; however, most existing methods for MPP are based solely on static molecular structures. In this study, we constructed multiple dynamic molecular datasets through MD simulations and incorporated molecular motion information into MPP tasks. We proposed a dynamic molecular representation method to effectively capture the key information embedded in molecular motion. Subsequently, these dynamic representations are applied to a deep learning-based prediction framework to perform MPP tasks. Experimental results show that incorporating MD information can significantly improve prediction performance. This study highlights the critical role of molecular motion in molecular property prediction and provides practical guidance for effectively leveraging dynamic information. The resources generated in this study, including MD datasets, DEMR models, and learning code, will support the sustained growth of computational drug discovery in the long term.

## 1 Introduction

**Fig 1:**
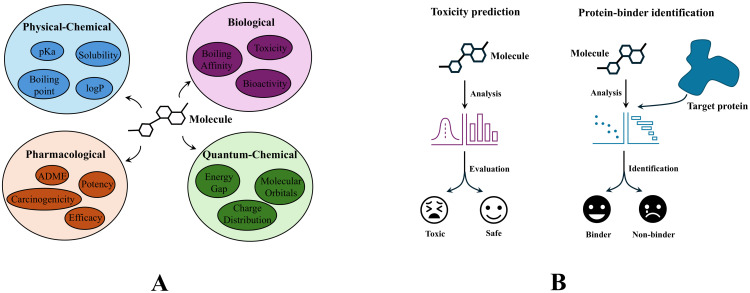
Introduction of molecular property prediction tasks. **A.** Diverse categories of molecular properties spanning chemical, biological, and pharmacological characteristics. **B.** Two representative biological property prediction tasks, including toxicity prediction and protein-binder identification.

Predicting molecular properties with high accuracy is a fundamental goal in drug discovery, computational chemistry, and chemical safety evaluation [1]. These molecular properties can span a wide spectrum of chemical, biological, and pharmacological characteristics of molecules (Fig 1A). In particular, biological properties such as toxicity, bioactivity and binding affinity play crucial roles in the development of new drugs [2], making in-silico prediction of these properties an active area of research.

*Toxicity prediction* and *protein-binder identification* are two representative tasks in computational drug discovery (Fig 1B). *Toxicity prediction* [3] has the goal of filtering out the compounds with potential safety issues before experimental testing, which helps reduce the failure rate of clinical trials. *Protein-binder identification* [4] aims to recognize small-molecule binders for a target protein, prioritizing them for downstream experimental validation. Aiming at these molecular property prediction (MPP) problems, a variety of computational approaches have been proposed in recent decades, with artificial intelligence (AI) methods emerging as a key focus among them.

During the application of AI techniques, particularly deep learning, to MPP tasks, molecules should be represented as a structured format suitable for computational learning. ***Molecular representation formats.*** In earlier studies, a molecule was frequently represented by a series of descriptors, showing its physical and chemical properties (e.g., molecular weight, logP, number of hydrogen-bond donors/acceptors, and polarizability) [5–7]. Another popular representation method is molecular fingerprints, such as extended-connectivity fingerprints (ECFPs). These fingerprints often adopt a binary string or a set of numbers to capture the structural patterns of a molecule [8–10]. In addition, representing a molecule as a sequence, such as a SMILES or SMARTS string [11–13], is another option for subsequent learning. A chemical string encodes molecular structures in a text-based format and is friendly to sequence-oriented learning. In recent years, molecules are often represented as graphs, with atoms treated as nodes and bonds as edges [14–16]. This representation method can store the natural structure and topological information of a molecule. ***Learning mechanisms.*** Treated as feature vectors, molecular descriptors and fingerprints have been extensively learned by traditional machine learning models (e.g., support vector machines and random forests) [9,17,18] and shallow neural networks (NNs) [19]. For chemical sequence representation, specialized large language models (LLMs) have been developed to learn tokenized sequence features [20–22]. A series of graph neural networks (GNNs) have also been devised to handle molecular graphs in MPP tasks [14–16,23,24]. Molecular geometric deep learning models, such as equivariant GNNs (EGNNs) [25] and ensemble conformational learning methods [26], have also been developed for MPP in recent years.

Despite the decent predictions, the earlier models mostly treat the input molecules as static. The molecular representations, typically generated from either a static molecular conformation or a fixed chemical sequence, are learned by machine learning models, LLMs, or GNNs. EGNNs take either a single molecular conformation or a set of conformations as input, with the primary focus of effectively representing and learning the given 3D molecular geometry. Although ensemble conformational learning methods account for the coexistence of multiple molecular conformations, the resulting “multi-conformation” representations are still essentially static and independent, lacking both temporal ordering and dynamic evolution information. Molecules are dynamic objects in solution, and their dynamics may drive the bioactivity and functions. Considering molecular dynamics (MD) is therefore an advantage in MPP tasks, but is limited to the lack of dynamics data in public repositories and the difficulty in learning such high-dimensional data. Consequently, this study aims to develop dynamics-enhanced models for MPP, with a focus on the two representative tasks of *toxicity prediction* and *protein-binder identification*. Our main contributions are listed below.

- Comprehensive MD simulations have been conducted on the molecules in several typical MPP sets, facilitating the use of dynamics data by the MPP community.
- Unlike existing methods that mainly rely on static 3D molecular conformations, this work explores the role of MD information in MPP task within deep learning frameworks.
- Different conformation sampling strategies have been investigated for dynamics-involved MPP tasks, providing practical guidance for the efficient use of such dynamics data.

## 2 Related work

In recent decades, AI-based methods have attracted considerable attention in MPP tasks. Early quantitative structure-activity relationship (QSAR) methods relied on manually crafted molecular descriptors/fingerprints (e.g. ECFPs) and were modeled using traditional machine learning algorithms [27]. They remain common baselines for evaluating the performance of MPP models [9,17,18].

With the development of deep learning, a variety of deep neural networks have been used for MPP tasks. Mayr et al. [28] proposed a deep neural network model (*DeepTOX*) for learning molecular fingerprints in MPP tasks, which achieved state-of-the-art performance at the time of its introduction. Convolutional Neural Networks (CNNs) are often used for image processing, but they have recently been introduced to molecular modeling and representation learning. The typical process involves encoding the sequence representation of molecules (e.g., SMILES strings) and applying convolutional operations to the extraction of underlying features [29,30]. In addition, the SMILES string of a molecule can be viewed as a form of chemical language, and many sequence-based models such as the *Transformer* architecture can be used for learning representations of such chemical languages [31]. Particularly, attempts have been made to use pre-trained LLMs (e.g. *ChemBERTa*) for MPP, leading to good predictive performance in benchmark MPP tasks [20–22].

Besides sequence representations, graph representations that capture molecular topological information are also popular in MPP tasks [16]. Molecules can be represented naturally as graph structures, where nodes correspond to atoms and edges represent chemical bonds between them. Based on such molecular graph representations, a series of MPP models have been developed using GNN architectures, addressing tasks such as *protein-binder identification* [32], *toxicity prediction* [16,33,34], *binding affinity prediction* [14,15,35] and more [36]. Ketkar et al. [16] evaluated the performance of benchmark GNN models on toxicity prediction tasks, demonstrating the effectiveness of GNNs in handling MPP tasks. Later, Cremer et al. [37] leveraged equivariant GNNs in toxicity prediction, and Xie et al. [38] introduced the *ToxKG* model by integrating heterogeneous knowledge graphs. Nguyen et al. [39] proposed the *GraphDTA* model to introduce molecular graph representations into drug-target binding affinity prediction, and subsequently Liu et al. [40] and Hou et al. [41] fed multimodal molecular data to GNNs for improved predictions. Overall, typical GNN architectures, such as GCN [42], GIN [43], GAT [44], GraphSAGE [45], and APPNP [46], have been frequently explored in MPP tasks. In addition, EGNNs [25] are designed to be equivariant to rigid transformations in 3D space, including rotations, translations, and reflections. When the input coordinates undergo such transformations, output geometric vectors (e.g., forces and displacements) transform accordingly, whereas scalar properties (e.g., energy) remain invariant. In contrast, ensemble conformational learning methods [26] explicitly leverage multiple molecular conformations by first generating a set of discrete, low-energy, and diverse conformers (e.g., using RDKit-based distance geometry), and then aggregating their features into molecular-level representations via permutation-invariant strategies such as pooling or attention. These methods have also been applied to MPP tasks in recent years.

## 3 Materials and methods

### 3.1 Generating MD data for MPP tasks

Before constructing dynamics-enhanced models for MPP tasks, we conducted reliable MD simulations on several widely applied benchmark datasets.

- *TOX21* from MoleculeNet are our base data for toxicity prediction. 12 different toxic effects of thousands of molecules were measured by specifically designed assays, constituting *TOX21*. These assays are either related to nuclear receptor signaling pathways (NR type) or cellular stress response pathways (SR type), demonstrating potential toxic effects of chemical compounds on different biological systems.
- *ADA17*, *EGFR* and *HIVPR* from DUD-E are the original data for protein-binder identification. *ADA17* includes a large group of small molecules that are either binders (actives) or non-binders (decoys) to ADAM Metallopeptidase Domain 17 (ADA17) protein. Similarly, *EGFR* and *HIVPR* indicate the molecules for epidermal growth factor receptor (EGFR) and HIV-1 protease (HIVPR), respectively.

The *GROMACS* [47] software package with *CGenFF* (CHARMM General Force Field) was primarily used to generate MD data. *CGenFF* is a widely used force field developed for biomolecules including proteins, nucleic acids, lipids, and small molecules. The total energy function in *CGenFF* consists of bonded terms and non-bonded terms, as shown below.


$$\begin{array}{cc} E_{\text{total}}=\; & E_{\text{bond}}+E_{\text{urey-bradley}}+E_{\text{angle}}\\ & +E_{\text{dihedral}}+E_{\text{improper}}+E_{\text{nonbonded}} \end{array}$$ (1)

where *E*_bond_, $E_{\text{urey-bradley}}$, *E*_angle_, *E*_dihedral_, *E*_improper_ and *E*_nonbonded_ represent the energy of bond stretching, Urey-Bradley, angle bending, dihedral torsion, improper torsion, and non-bonded (van der Waals and Electrostatics), respectively. For atoms, *CGenFF* defines an extensive set based on atomic type (e.g., *C*, *H*, *O*, *N*, *S*, *P*, etc.), hybridization state (e.g., sp^3^, sp^2^, aromatic), and bonding context. In this study, we used *CGenFF* to generate the topologies of molecules through an online interface (https://cgenff.com/). Due to non-standard or low-quality inputs, *CGenFF* may fail to parameterize certain molecules, which were excluded from our MD datasets to ensure reliable MD simulations. Detailed exclusion criteria include: ① those whose initial structures are of poor quality, ② those failing to generate MD-required topological files, and ③ those containing atoms that cannot be recognized by *CGenFF*.

**Fig 2:**
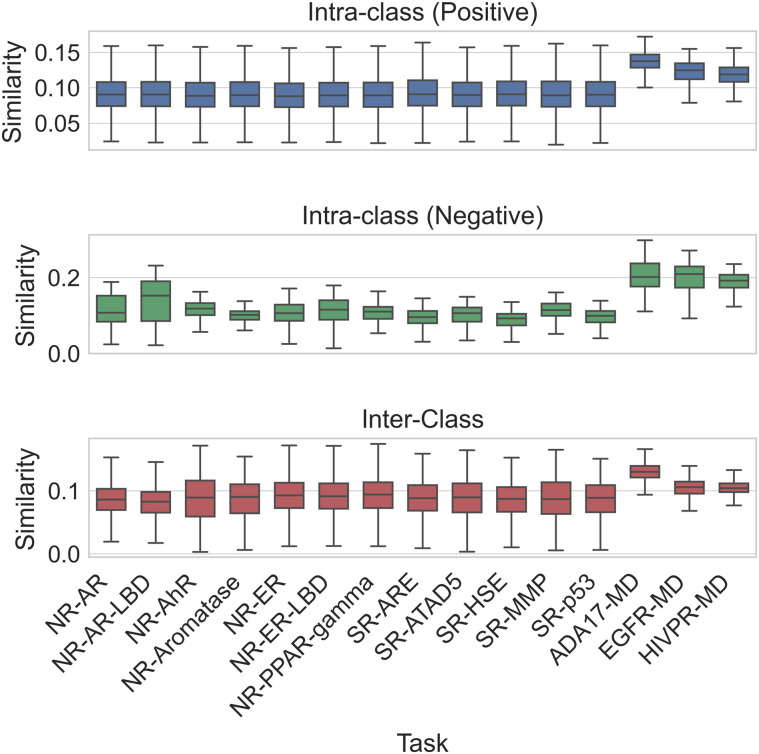
Pairwise similarity for molecules in each filtered dataset.

Finally, the filtered datasets for each MPP task are presented in Table 1. We name these datasets as “TOX21-MD” for toxicity prediction and ” DUD-E-MD” for protein-binder identification. To verify the diversity of the filtered datasets, we examined the average pairwise molecular similarity within each class (e.g., positive or negative) and between classes (e.g., positive vs. negative) in each set. The similarity is defined as the average *Tanimoto similarity* of the *Morgan fingerprints* for molecule pairs. Specifically, the *Morgan fingerprint* of each molecule was generated using the RDKit package from its *SMILES* representation, with default parameters (radius = 2, length = 2048). *Tanimoto* on *Morgan fingerprints* quantifies how similar two molecular structures are in terms of their substructures. As shown in Fig 2, both positive and negative molecules exhibit low intra-class similarity, suggesting high structural heterogeneity within each class. The low inter-class similarity between positive and negative molecules indicates that the structural patterns distinguishing the two classes do not substantially overlap.

**Table 1:** Statistics on our MD Datasets for MPP tasks.

Task Category	Task Name	Number of molecules (positive/negative)	Imbalance Ratio
Toxicity prediction (TOX21-MD)	NR-AR	4156 (179/3977)	1:22.2
	NR-AR-LBD	6758 (117/3818)	1:32.6
	NR-AhR	3774 (472/3302)	1:7.0
	NR-Aromatase	3347 (134/3213)	1:24.0
	NR-ER	3644 (500/3144)	1:6.3
	NR-ER-LBD	4017 (212/3805)	1:17.9
	NR-PPAR-gamma	3739 (105/3634)	1:34.6
	SR-ARE	3488 (508/2980)	1:5.8
	SR-ATAD5	4111 (165/3946)	1:23.9
	SR-HSE	3820 (191/3629)	1:19.0
	SR-MMP	3368 (499/2869)	1:5.7
	SR-p53	3946 (224/3722)	1:16.6
Protein-binder identification (DUD-E-MD)	ADA17	1176(420/756)	1:1.80
	EGFR	1485(436/1049)	1:2.41
	HIVPR	994(385/609)	1:1.58

**Fig 3:**
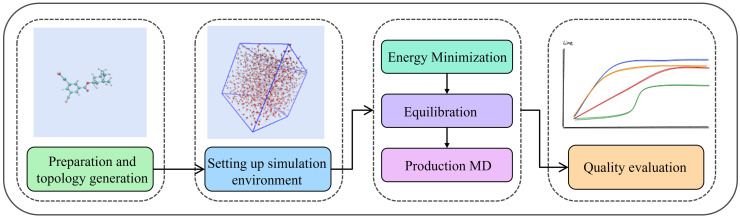
A general workflow for MD simulations.

Next, we performed MD simulations on the molecules in ” TOX21-MD” and ” DUD-E-MD” following the workflow shown in Fig 3. The MD implementation details are described below.

(1) **Preparation and topology generation.** Generating the 3D structure (in ‘pdb’ or ‘mol2’ format) of a molecule from its SMILES string is the starting point and can be accomplished using the *RDKit* software package. Based on the 3D structure, a molecule can be parameterized using *GROMACS*, and its topological files were generated via the *CGenFF* Web Server.
(2) **Setting up the simulation environment.** Since molecules are dynamic in solution, we need to set up a solution environment computationally before the simulations. For each small molecule, a rectangular simulation box was defined, sharing the same center as the molecule and extending at least 1.0 *nm* beyond the molecule in all directions. Subsequently, the molecule was computationally solvated, with the *SPC* water model applied. This water environment is the main simulation environment.
(3) **Energy Minimization (EM).** EM is performed to eliminate unfavorable atomic contacts and high-energy conformations. 50,000 minimization steps using the steepest descent algorithm were performed on each system, with the maximum force on the system often reduced to below 1000 *kJ*/*mol*/*nm*. The Particle Mesh Ewald (PME) method was employed to treat long-range electrostatic interactions, with the van der Waals force cutoff distance set to 1.2 *nm* and periodic boundary conditions applied in all directions. This procedure ensures a stable initial conformation for the system.
(4) **System equilibration.** This process includes both NVT and NPT equilibration phases, which aim to stabilize the system at the target temperature and pressure. *NVT equilibration:* The system temperature was equilibrated to 300 *K* using the V-rescale thermostat method for 100 *ps* (time step of 0.002 *ps* and total 50000 steps) with a leap-frog integrator. Positional restraints were applied to the molecules, and neighbor searching was performed using the Verlet cut-off scheme (1.2 *nm* cut-off for non-bonded interactions). *NPT equilibration:* Subsequently, the NPT equilibration for 100 *ps* was performed using the Berendsen barostat method to regulate the pressure to 1 *bar*, with an isothermal compressibility of 4.5 $\times 10^{-5}$ *bar*^-1^ and the same positional restraints. The temperature control parameters were kept consistent with those in the NVT phase.
(5) **Production MD.** After removing the positional restraints, an all-atom molecular dynamics simulation was performed for 10 *ns* using a leap-frog integrator (time step of 2 *fs*, totaling 5000000 steps). The system temperature and pressure were maintained. Non-bonded interactions were treated with a cut-off distance of 1.2 *nm*. Electrostatic interactions were calculated using the PME method, and van der Waals interactions were corrected with the force-switch algorithm. Periodic boundary conditions were applied in all three spatial dimensions, and dispersion correction was enabled to improve the accuracy of energy and pressure calculations. The MD trajectories were saved every 1 *ps* during the simulation for each system.
(6) **Quality evaluation.** Due to the use of periodic boundary conditions, molecules may appear ‘broken’ or may ‘jump’ back and forth across the box during the simulation. Therefore, trajectory post-processing was performed to re-center the molecules and restore the integrity of the unit cell. To evaluate the quality of the MD simulations, we examined the temperature (NVT phase), density (NPT phase), total energy (production MD phase), and root-mean-square deviation (RMSD, production MD phase) of each system. The stable trends of these indicators confirm the reliability and convergence of the MD simulation process.

### 3.2 Dynamics-enhanced molecular representation modeling

Molecular representations play a crucial role in MPP tasks, and conventional methods often overlook the dynamic nature of molecules. In this study, we introduce a dynamics-enhanced molecular representation (DEMR) method, which describes the structures and dynamics of molecules. Compared with representations relying solely on static molecular structures, DEMR substantially enhances the expressive power and informativeness of molecular features.

Through MD simulations, we collected a dynamics trajectory spanning 10 *ns* for each molecule. Such a trajectory contains 10000 frames (structural conformations), and can be denoted as $\left\{{\text{c}}_{1}^{i},\ldots ,{\text{c}}_{10,000}^{i}\right\}$ for the *i*-th molecule. The frames were recorded in relatively short time intervals (1 *ps*) because our objective was to examine the impact of the frequency-sampling strategy on the subsequent learning process. Given a molecular trajectory, *representing each frame* ${\text{c}}_{j}^{i}$ and *sampling the frames further* are the two key points of DEMR.

#### 3.2.1 Representing each frame${𝐜}_{j}^{i}$.

To efficiently capture the structural information of each frame, both the atomic identities and their relative spatial positions are essential. Considering the cost of learning models, time-series models based on geometric or graph encoders typically have high complexity and require significant hardware resources. Inspired by Zheng et al. [48], we used an atom-type-based and distance-guided strategy to represent each molecular frame. This approach ensures that the model efficiently and accurately captures the dynamic details of molecules. First, we defined the following set of atom types.


$$\begin{array}{c} {ℒ}_{AT}=\{\text{C},\text{N},\text{O},\text{H},\text{P},\text{S},\text{HAX},\text{DU}\} \end{array}$$ (2)

*HAX* refers to halogen elements (*F*, *Cl*, *Br*, and *I*) and *DU* represents all remaining elements. Further, a list of atom-pair types can be defined as a matrix.


$$\begin{array}{cc} {ℳ}_{APT} & ={ℒ}_{AT}\times {ℒ}_{AT}^{T}\\ & ={\left[\begin{array}{ccc} \left(\text{C},\text{C}\right) & \cdots & \left(\text{C},\text{DU}\right)\\ \vdots & \ddots & \vdots \\ \left(\text{DU},\text{C}\right) & \cdots & \left(\text{DU},\text{DU}\right) \end{array}\right]}_{8\times 8} \end{array}$$ (3)

With respect to atomic interactions, we consider the following distance ranges.


$$\begin{array}{c} {ℒ}_{DR}=\{rg_{1},rg_{2},rg_{3}\}=\{[1.0,2.0),[2.0,3.0),[3.0,4.0)\} \end{array}$$ (4)

These ranges are defined to encode short-, medium-, and long-range spatial interactions in molecular systems. Specifically, short-range interactions mostly correspond to covalent bonds; medium-range interactions are typically associated with hydrogen bonds and dipole-dipole interactions, and long-range interactions reflect van der Waals forces and hydrophobic contacts [49]. Based on this scheme, a feature representation can be derived for each frame ${\text{c}}_{j}^{i}$ as follows.


$$\begin{array}{cccc} & 𝐗=[\begin{array}{ccc} x_{\left(\text{C},\text{C}\right)}^{rg1} & \cdots & x_{\left(\text{C},\text{DU}\right)}^{rg1}\\ \vdots & \ddots & \vdots \\ x_{\left(\text{DU},\text{C}\right)}^{rg1} & \cdots & x_{\left(\text{DU},\text{DU}\right)}^{rg1} \end{array}] & \left[\begin{array}{ccc} x_{\left(\text{C},\text{C}\right)}^{rg2} & \cdots & x_{\left(\text{C},\text{DU}\right)}^{rg2}\\ \vdots & \ddots & \vdots \\ x_{\left(\text{DU},\text{C}\right)}^{rg2} & \cdots & x_{\left(\text{DU},\text{DU}\right)}^{rg2} \end{array}\right] & \left[\begin{array}{ccc} x_{\left(\text{C},\text{C}\right)}^{rg3} & \cdots & x_{\left(\text{C},\text{DU}\right)}^{rg3}\\ \vdots & \ddots & \vdots \\ x_{\left(\text{DU},\text{C}\right)}^{rg3} & \cdots & x_{\left(\text{DU},\text{DU}\right)}^{rg3} \end{array}\right] \end{array}$$ (5)

Here $x_{\left(s,t\right)}^{rg_{k}}$ denotes the count of atom pairs of type (*s*,*t*) in Eq. 3, whose interatomic distance falls wi*t*hin the range $rg_{k}$. The resulting feature tensor, $𝐗\in {\mathbb{R}}^{3\times 8\times 8}$, efficiently encodes the key atomic and structural information of the frame. In addition to this general scheme, specific atom-type pairs beyond those in Equation 3 and distance ranges outside those in Equation 4 can be designed in various application scenarios.

#### 3.2.2 Sampling the frames further.

Originally, we collected *J* = 10,000 frames $\{{\text{c}}_{j}^{i}{\}}_{j=1}^{J}$ for each molecule, and each frame can be represented as ${𝐗}_{j}^{i}$ according to Eq. 5. To leverage the dynamics information more efficiently, we construct a ${DEMR}^{i}$ tensor as an enriched representation based on different sampling strategies. ① Time-interval sampling (DEMR-TIS). Different intervals along the time axis were used to sample the frames. A series of time intervals Freq∈{1 *ps*, 2 *ps*, 5 *ps*, 10 *ps*, 20 *ps*, 50 *ps*, 100 *ps*, 200 *ps*, 500 *ps*, 1000 *ps*} were adopted in this study, and $T=\frac{10,000}{Freq}$ frames were sampled. ② RMSD-based sampling (DEMR-RBS). Frames were ranked according to their RMSD values relative to the original conformation and categorized into high-, medium-, and low-level changes (HLC, MLC, and LLC). Focusing on a category (HLC, MLC or LLC), *T* frames were sampled, with *T* selected from {2,5,10,20,50,100,1000}. These sampled frames were weighted by their RMSD values during the subsequent learning process. This strategy was designed to evaluate the effect of molecular conformational variation on model performance. ③ Hybrid-category sampling (DEMR-HCS). To jointly consider all three categories (HLC, MLC, and LLC), $\frac{T}{3}$ frames were sampled from each category and combined into a set of *T* frames. Learnable weights were then assigned to these frames in the following learning stage to assess the relative importance of different conformational categories for the MPP tasks. Finally, the newly sampled *T* frames yield an enriched representation of the *i*-th molecule as follows:


$$\begin{array}{c} {DEMR}^{i}=[\begin{array}{c} {𝐗}_{j_{1}}^{i},\ldots ,{𝐗}_{j_{T}}^{i} \end{array}] \end{array}$$ (6)

This tensor captures both the static structural features and the dynamic conformational changes of the molecule, providing richer information for developing learning models in MPP tasks.

### 3.3 Dynamics-involved learning architecture design

**Fig 4:**
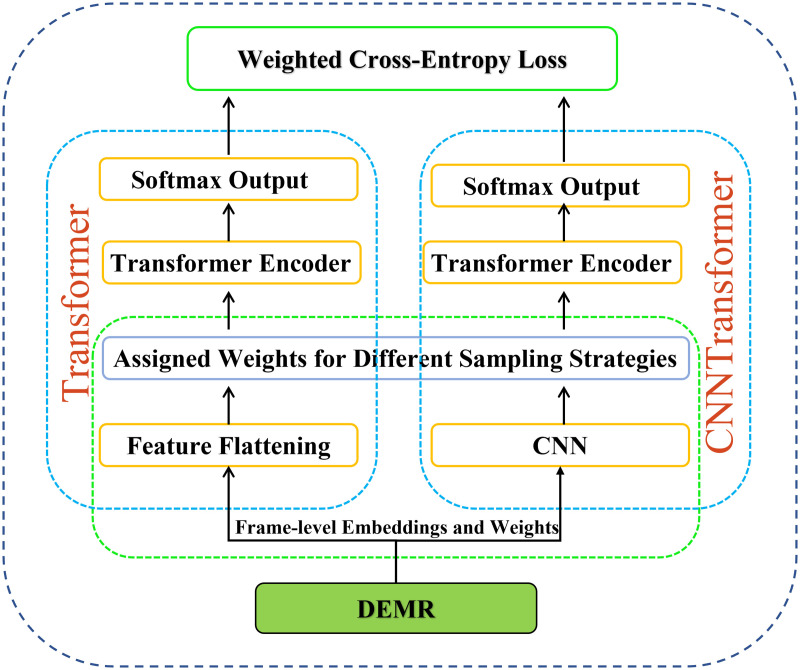
The network architecture for dynamics-enhanced MPP tasks.

To effectively learn the **DEMR** tensors, we design several hybrid neural network architectures, as illustrated in Fig 4.

#### 3.3.1 Frame-level embeddings and weights.

The input tensor $DEMR\in {\mathbb{R}}^{T\times 3\times 8\times 8}$ consists of *T* structural frames with each frame encoding the co-occurrence of atom type pairs across three distance intervals. The sample index *i* is omitted now for simplicity. For each frame, we map it into an embedding space using two alternative strategies: a *CNN layer* or a *flattening layer*. *The CNN layer.* This module processes each frame and projects it into a fixed-dimensional embedding vector of size out_dim.


$$\begin{array}{c} H_{j_{t}}=f_{\text{CNN}}({DEMR}_{j_{t}}) \end{array}$$ (7)

Here, $𝐇\in {\mathbb{R}}^{T\times out\_dim}$ denotes the output of this module, and *f*_CNN_ represents the CNN layer. *The flattening layer.* The feature tensor ${𝐗}_{j_{t}}\in {\mathbb{R}}^{3\times 8\times 8}$ corresponding to a specific frame is directly flattened into a vector of length 192, as follows.


$$\begin{array}{c} H_{j_{t}}=f_{\text{flat}}({DEMR}_{j_{t}}) \end{array}$$ (8)

The feature-handling strategies described above were combined with three frame-sampling strategies (Section 3.2), with different weights assigned to the sampled frames, to generate the overall embedding for subsequent learning. ① DEMR-TIS. *T* frames were sampled at different time intervals, and all frames were assigned equal weights. ② DEMR-RBS. *T* frames were sampled based on their RMSD ranges, each corresponding to a single RMSD category (HLC, MLC, or LLC). These frames were weighted according to their RMSD values. ③ DEMR-HCS. *T* frames covering all three RMSD categories (HLC, MLC, and LLC) were sampled, and learnable weights were assigned to them to investigate the respective contributions of different RMSD categories to the prediction tasks. Combining these three scenarios, the weights for the selected *T* frames are listed as below.


$$\begin{array}{c} w_{t}=\left\{ \begin{array}{cc} 1, & \text{if sampling by DEMR-TIS},\\ {RMSD}_{j_{t}}, & \text{if sampling by DEMR-RBS},\\ f_{w}({𝐇}_{j_{t}}), & \text{if sampling by DEMR-HCS}. \end{array}\right. \end{array}$$ (9)

Finally, concatenating (||) the weighted frame-level embeddings yields the overall embedding for further learning.


$$\begin{array}{c} \hat{𝐇}=\underset{t=1}{\overset{T}{\left|\right|}}w_{t}\cdot H_{j_{t}} \end{array}$$ (10)

where $\hat{𝐇}$ denotes the weighted result that has the same dimensionality as ${𝐇}_{j_{t}}$.

#### 3.3.2 Transformer encoder.

This module was employed to process $\hat{𝐇}$ and capture complex interactions among different frames, which are essential for learning high-level dynamics representations.


$$\begin{array}{cc} & 𝐙=f_{\text{Trans}}(\hat{𝐇}) \end{array}$$ (11)

where **Z** represents the output of the Transformer module. *f*_Trans_ denote the functions of the Transformer encoder module.

#### 3.3.3 Softmax output.

Each MPP task was formulated as a binary classification problem (toxic vs. non-toxic or binder vs. non-binder). The final molecular representation **Z** was then passed through a multi-layer perceptron (MLP), and the resulting logits were normalized using the softmax function to produce class probabilities:


$$\begin{array}{cc} 𝐳 & =f_{\text{MLP}}(𝐙)\\ P_{c} & =\frac{e^{z_{c}}}{\sum_{a=1}^{2}e^{z_{a}}},c=1,2 \end{array}$$ (12)

where $𝐳=[z_{1},z_{2}]\in {\mathbb{R}}^{1\times 2}$ represents the output of the MLP, with *z*_1_ and *z*_2_ indicating the unnormalized logits for the two classes. The function *f*_MLP_ denotes the transformation implemented by the MLP. $P_{c}$ is the predicted probability of the molecule belonging to class *c* (i.e., positive or negative).

#### 3.3.4 Weighted cross-entropy loss.

As shown in Table 1, class imbalance is evident in MPP tasks. This imbalance can lead to biased predictions and should be carefully addressed. In this study, we incorporated a weighted cross-entropy loss (WCEL) function into the training process. The weights were calculated based on the number of samples in each class, with higher weights assigned to the positive class. This allows the model to focus on the minority positive class, thereby enhancing overall classification performance.


$$\begin{array}{c} LS=-\frac{1}{N_{s}}\sum_{n=1}^{N_{s}}\sum_{c=1}^{2}w_{c}\;y_{n,c}\log\left(\frac{e^{z_{n,c}}}{\sum_{a=1}^{2}e^{z_{n,a}}}\right) \end{array}$$ (13)

where LS denotes the loss function, $N_{s}$ is the number of samples, and *y* represents the ground truth after one-hot encoding; $y_{n,c}$ is the label corresponding to the *c*-th class of the *n*-th sample, and $z_{n,c}$ is the model output for the *c*-th class of the *n*-th sample; $w_{c}$ is the weight of *c*-th class, defined as $w_{c}=\frac{N_{s}}{2N_{c}}$, $N_{c}$ denotes the number of samples belonging to the *c*-th class.

## 4 Experiments and results

### 4.1 Generated MD datasets

According to the workflow in Fig 3, all-atom MD simulations were conducted for 8,145 molecules for MPP tasks. These MD simulations were performed on high-performance computing servers equipped with NVIDIA V100 32GB GPUs. The molecules in our dataset contain between 4 and 197 atoms, resulting in varying computational costs for simulations.

To ensure reliable MD simulations, we monitored the trends of several metrics (temperature, density, total energy and RMSD) during the MD simulations. The quality of three representative MD systems, with varying sizes of molecules (Table 2), were evaluated. With increasing molecular size, MD simulations require more time and computational resources, as expected.

**Table 2:** Representative MD systems for evaluation.

Dataset	Molecule ID	Size of molecule	MD time cost (seconds)
TOX21-MD	TOX20800	25	640.7
DUD-E-MD (ADA17)	decoymol3153	58	1724.6
DUD-E-MD (EGFR)	decoymol6361	101	5323.7

**Fig 5:**
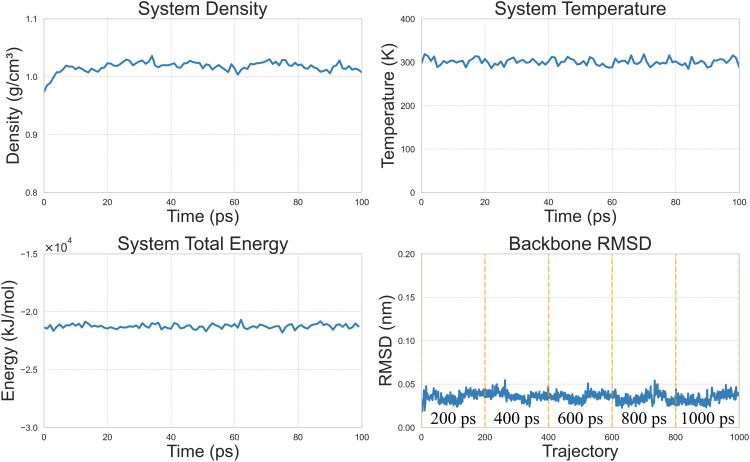
Quality evaluation for a representative MD system (molecule ID: TOX20800, size of molecule: 25 atoms, dataset: TOX21-MD).

**Fig 6:**
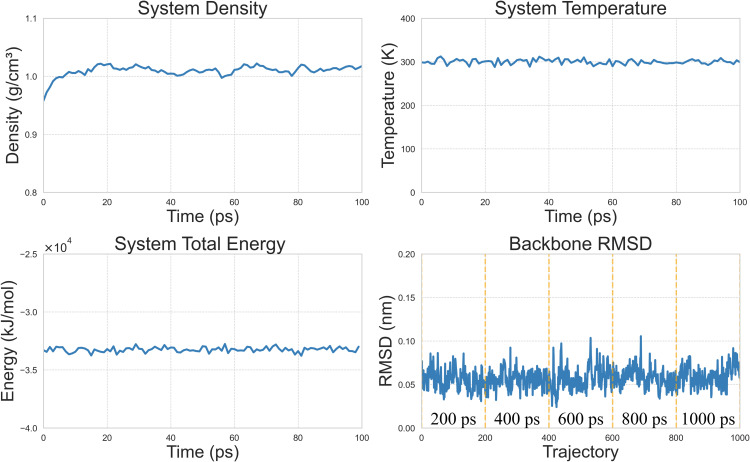
Quality evaluation for a representative MD system (molecule ID: decoymol3153, size of molecule: 58 atoms, dataset: DUD-E-MD (ADA17 task)).

**Fig 7:**
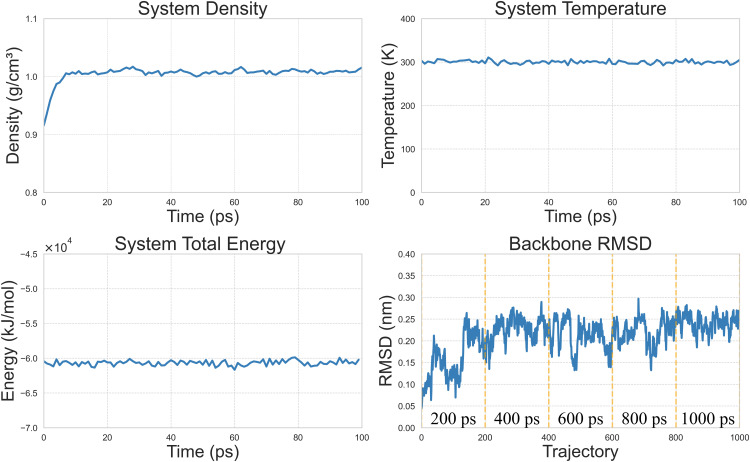
Quality evaluation for a representative MD system (molecule ID: decoymol6361, size of molecule: 101 atoms, dataset: DUD-E-MD (EGFR task)).

For each system, the temperature, density, total energy and backbone RMSD over time are shown in Figs 5–7. These results confirmed that the molecular structures remained structurally stable and well equilibrated throughout the MD simulations. To facilitate the use of our generated MD data by the public, all the data have been deposited in a *Zenodo* repository at https://doi.org/10.5281/zenodo.15788151. To date, our MD datasets have reached hundreds of downloads on *Zenodo*.

### 4.2 Experimental configuration for learning models

**Fig 8:**
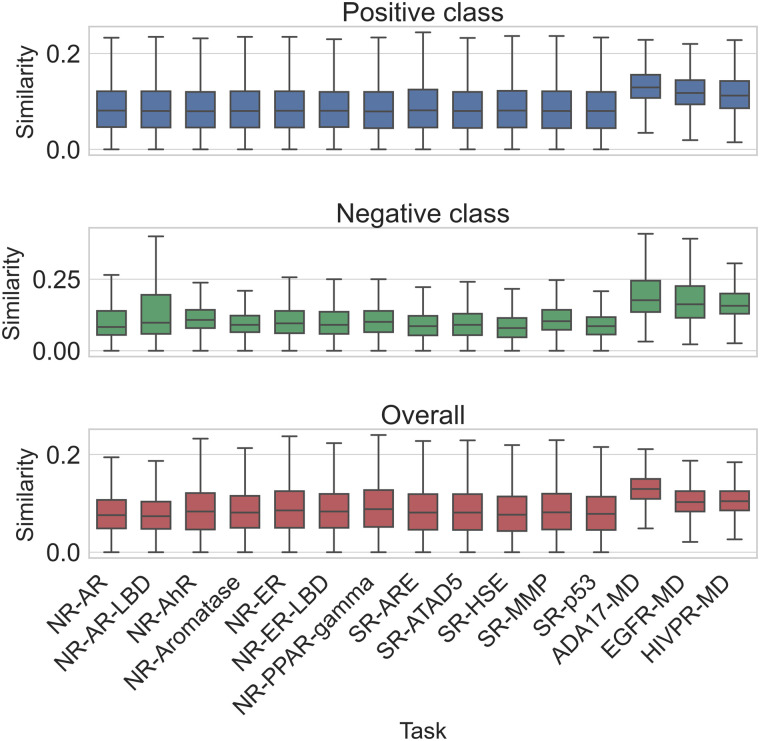
Pairwise similarity of molecules between the training and test sets.

All the models were trained on NVIDIA A100 GPUs. For each task, the dataset was split into training, validation, and test sets with a ratio of 7:1:2, using a batch size of 32 and 200 training epochs. It is important to note that the datasets were split by molecule; therefore, all conformations of the same molecule were assigned to the same set (either training or test). Furthermore, we calculated the molecular similarity between the training and test sets as a similarity control, and the results are shown in Fig 8. The results show that the average similarity between the training and test sets is low, whether considering the positive class, the negative class, or all samples. To prevent overfitting and enhance generalization, an early stopping strategy with a patience of 10 epochs was employed. Due to the class imbalance, model performance was evaluated using ROC-AUC, Balanced Accuracy (Balanced Acc), and Matthews Correlation Coefficient (MCC).

#### 4.2.1 Dynamics-involved learning.

The *CNN module* comprises two 2D convolutional layers with 3×3 kernels. The ReLU activation function is applied after each layer, and dropout is used to prevent overfitting. The CNN output is projected to a 128-dimensional vector (out_dim=128) via a fully connected layer. The *Transformer module* consists of 6 encoder layers, and each encoder layer contains 4 self-attention heads. Learnable positional encodings are introduced to capture temporal dependencies more effectively. The classifier has a 2-layer MLP with ReLU activation and Dropout. The final binary classification probabilities are obtained via a *Softmax layer*, facilitating effective discrimination of molecular properties.

#### 4.2.2 Baselines.

We compared our models with a variety commonly used methods in MPP tasks. ① Some of them employ molecular fingerprints (e.g., ECFP and MACCS) as feature representations, and adopt traditional machine learning models for MPP. Four machine learning models were considered, including Decision Tree (DT), K-Nearest Neighbor (KNN), Logistic Regression (LR), and Naive Bayes (NB). ② The *DeepTox* model leverages ECFPs and Deep Neural Networks (DNNs) is often regarded as a modern benchmark for toxicity prediction. Here we also included it in the baseline list. ③ Graph-based models serve as strong competitors in MPP tasks, and thus we added them as another set of baselines. Here each molecule is represented as a graph, with the atoms as nodes and the bonds as edges. Basic atomic properties (e.g., atom types) were used to characterize the nodes. Five graph neural network-based models (APPNP, GAT, GCN, GIN, and GraphSAGE) were adopted to learn these molecular graphs and make predictions. ④ Treating the molecules as SMILES sequences, a pre-trained large language model (ChemBERTa) was employed to extract feature embeddings, which were then fed into a DNN for MPP. ⑤ A CNN model using a single static 3D molecular conformation was adopted to examine the impact of dynamic information in MPP tasks. The 3D atomic coordinates of the molecules in their initial MD simulation states were fed into the CNN for predictive modeling. ⑥ We added a baseline using a modern EGNN with full 3D atomic coordinates, with the additional goal of verifying the importance of dynamics information. The 3D atomic coordinates of the molecules in their initial MD simulation states, optimized with *MMFF*/*UFF*, were fed into the EGNNs for predictive modeling. The EGNN model consisted of four EGNN layers with a hidden dimension of 128.

### 4.3 Results and analysis

#### 4.3.1 Overall performance.

Table 3 summarizes the results of both the baselines and our proposed models (Fig 4). For each task, experiments were conducted over three independent runs with different random seeds, and the mean and standard deviation across runs were reported. Moreover, we averaged the results across all tasks in *toxicity prediction (TOX21-MD)* and *protein-binder identification (DUD-E-MD)* to provide a clearer overall performance comparison. The results show that the *toxicity prediction* tasks (most models achieve an ROC-AUC below 0.75) are more challenging than the *protein-binder identification* tasks (most models achieve an ROC-AUC above 0.95). It is partly due to that the *toxicity prediction* sets used in this study are larger and more imbalanced (Table 1). However, our models achieved better performance, underscoring their robustness in handling large and imbalanced datasets. Although achieving comparable performance with most of deep-learning models (GNNs and *ChemBERTa*), our models underperform the other baselines in the *protein-binder identification* tasks. This may be largely due to the limited number of molecules in these sets (Table 1).

**Table 3:** Model performance comparison for MPP tasks.

Feature	Model	TOX21-MD			DUD-E-MD		
		ROC-AUC	Balanced Acc	MCC	ROC-AUC	Balanced Acc	MCC
ECFP	DT	0.631±0.062	0.625±0.065	0.257±0.123	0.969±0.026	0.969±0.028	0.937±0.051
	KNN	0.679±0.062	0.554±0.055	0.199±0.180	0.868±0.087	0.634±0.041	0.352±0.023
	LR	0.732±0.073	0.628±0.052	0.285±0.102	0.998±0.001	0.989±0.005	0.982±0.007
	NB	0.564±0.051	0.564±0.051	0.094±0.076	0.805±0.054	0.805±0.054	0.678±0.051
MACCS	DT	0.647±0.056	0.632±0.060	0.253±0.114	0.947±0.027	0.947±0.027	0.891±0.059
	KNN	0.734±0.070	0.605±0.073	0.304±0.169	0.984±0.014	0.947±0.022	0.889±0.049
	LR	0.764±0.064	0.603±0.069	0.284±0.169	0.996±0.003	0.981±0.014	0.954±0.034
	NB	0.564±0.066	0.529±0.043	0.046±0.064	0.887±0.095	0.854±0.073	0.704±0.139
ECFP	DeepTox	0.767±0.059	0.627±0.073	0.359±0.156	0.998±0.002	0.992±0.008	0.985±0.013
Graph	APPNP	0.711±0.051	0.502±0.006	0.009±0.027	0.921±0.023	0.799±0.049	0.626±0.085
	GAT	0.731±0.055	0.516±0.032	0.062±0.108	0.992±0.006	0.950±0.033	0.914±0.055
	GCN	0.730±0.059	0.516±0.033	0.060±0.109	0.982±0.007	0.941±0.027	0.891±0.049
	GIN	0.756±0.076	0.559±0.070	0.187±0.189	0.989±0.005	0.967±0.021	0.938±0.039
	GraphSAGE	0.748±0.057	0.527±0.047	0.101±0.134	0.988±0.009	0.943±0.033	0.900±0.059
Sequence	ChemBERTa	0.736±0.059	0.553±0.082	0.206±0.138	0.974±0.020	0.917±0.047	0.845±0.078
3D coordinates	EGNN	0.748±0.054	0.576±0.096	0.193±0.159	0.991±0.006	0.955±0.027	0.823±0.036
	CNN	0.678±0.045	0.604±0.046	0.307±0.110	0.987±0.003	0.948±0.017	0.889±0.034
DEMR-TIS	Transformer	0.813±0.017	0.73±0.020	0.339±0.040	0.995±0.001	0.967±0.006	0.925±0.013
	CNNTransformer	0.760±0.032	0.670±0.052	0.290±0.028	0.989±0.002	0.949±0.011	0.885±0.032
DEMR-RBS	Transformer	0.811±0.009	0.726±0.018	0.330±0.035	0.993±0.001	0.958±0.008	0.901±0.024
	CNNTransformer	0.702±0.037	0.616±0.053	0.192±0.040	0.982±0.004	0.938±0.010	0.857±0.024
DEMR-HCS	Transformer	0.809 ±0.009	0.726±0.021	0.317±0.036	0.993±0.003	0.958±0.018	0.899±0.047
	CNNTransformer	0.708±0.040	0.611±0.076	0.209±0.039	0.983±0.007	0.962±0.011	0.884±0.027

Notably, using only flattened features (*Transformer* model), rather than CNN-processed features (*CNNTransformer* model), leads to even more promising results. This may stem from the lack of local spatial correlations among atom-type pairs, reducing the usefulness of convolutions for processing them. The discriminative information in DEMR features is often distributed across global interaction patterns, potentially reflecting weaker local interactions.

**Fig 9:**
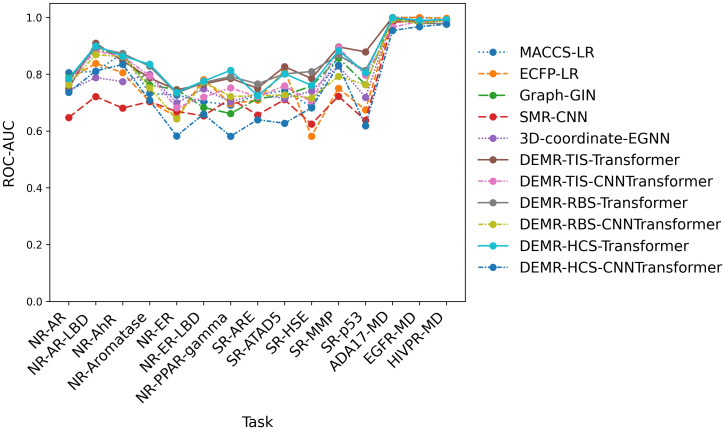
Performance evaluation for toxicity prediction tasks.

To further demonstrate the effectiveness of our approach, we analyzed the ROC-AUC metrics for all *toxicity prediction* tasks, as shown in Fig 9. Several representative baseline models, including LR trained with MACCS and ECFP, GIN, EGNN and CNN using 3D coordinates, were evaluated alongside our proposed methods. Together with the results reported in Table 3, these findings indicate that the DEMR-based Transformers achieves better performance than CNN and EGNN models using full 3D coordinates features. This again suggests that the performance improvements are driven by dynamics information rather than by the use of static 3D molecular information. On average, the *Transformer* model using DEMR-TIS or DEMR-HCS achieve the best performance across most tasks. Moreover, NR-AR-LBD is a well-known challenging task in the field of *toxicity prediction* [50], and our models achieved competitive results.

#### 4.3.2 Sampling strategies analysis.

To systematically evaluate the influence of MD sampling schemes on DEMR construction, we investigated three sampling strategies from the perspectives of temporal resolution, conformational diversity, and model sensitivity, respectively.

**Fig 10:**
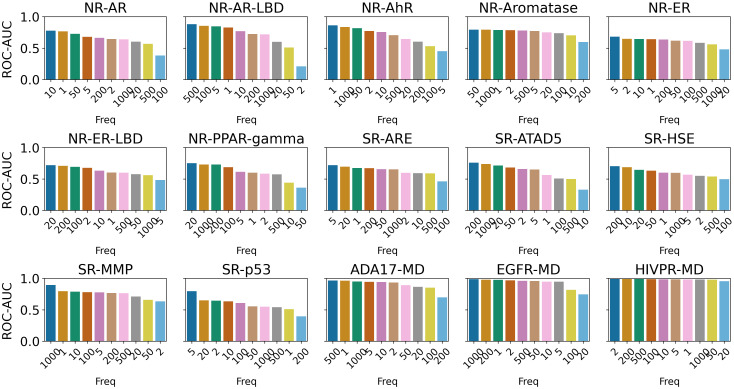
ROC-AUC performance of our models on different datasets. CNNTransformer were adopted to learn DEMR-TIS representations.

**Fig 11:**
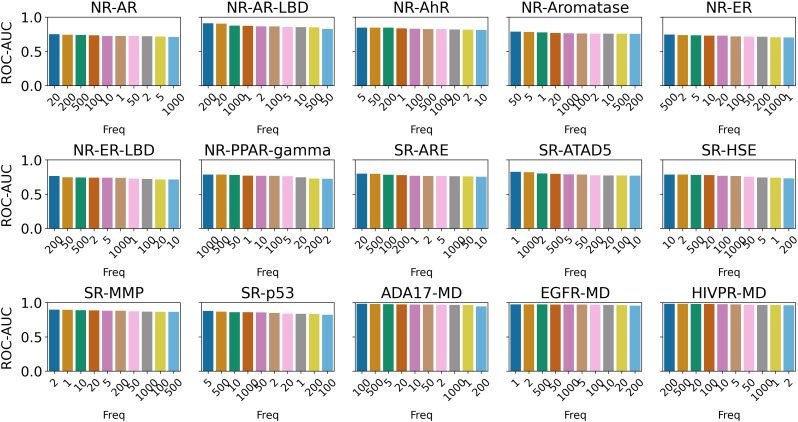
ROC-AUC performance of our models on different datasets. Transformer were adopted to learn DEMR-TIS representations.

**Fig 12:**
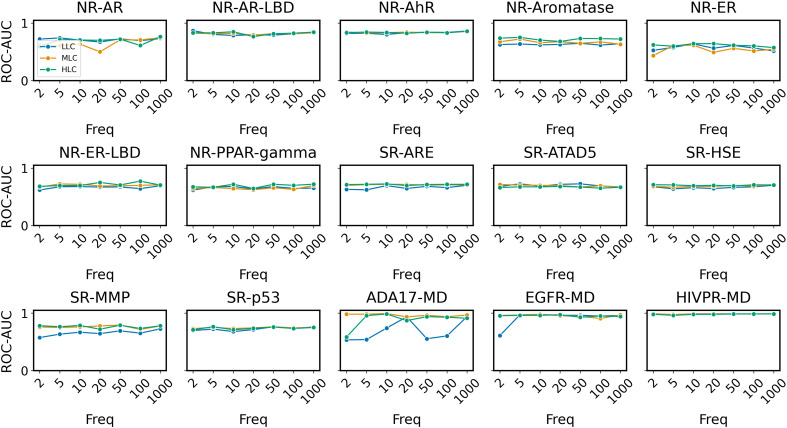
ROC-AUC performance of our models on different datasets. CNNTransformer were adopted to learn DEMR-RBS representations.

**Fig 13:**
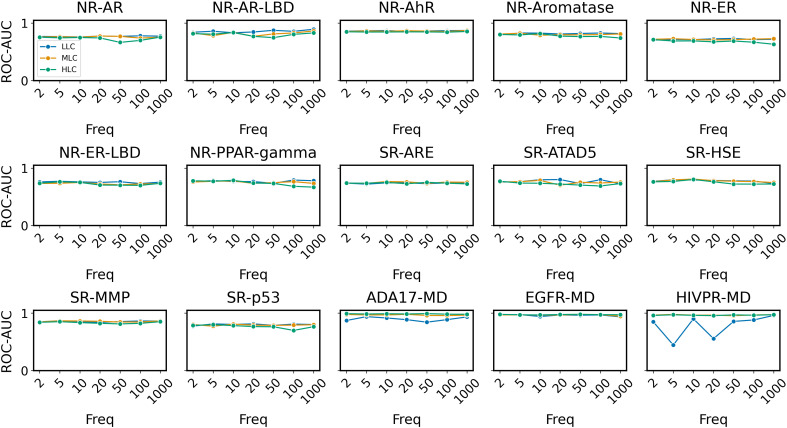
ROC-AUC performance of our models on different datasets. Transformer were adopted to learn DEMR-RBS representations.

- *DEMR-TIS: Sampling Frequency Analysis.* We first examined the effect of temporal sampling intervals *Freq* on MPP performance. Figs 10 and 11 present the average ROC-AUC results of using *CNNTransformer* and *Transformer* to process the DEMR representations, respectively. The results show that model performance is highly sensitive to sampling frequency and selecting task-specific sampling frequencies may improve the predictive accuracy. In other words, selecting task-specific sampling frequency is critical for effectively leveraging MD-enhanced features.
- *DEMR-RBS: Conformational Diversity Analysis.* We further investigated the effect of conformational diversity by constructing DEMR representations from three RMSD-based regions, including HLC, MLC, and LLC. Figs 12 and 13 shows the ROC-AUC results under these settings. The conformations of HLC, MLC or LLC were ranked based on the results of the Backbone RMSD. The models trained on frames located in the HLC and MLC regions generally outperform those trained on frames located in the LLC region. In particular, performance degradation is observed in several datasets when only LLC-conformations are used. It indicates that conformations with low structural deviation from the reference structure provide insufficient structural variability, leading to the limited capture of functionally relevant molecular states. While the HLC region introduces greater structural diversity, it may include extreme molecular conformations. The MLC region provides a balance between structural variation and stability, often leading to consistently strong performance across tasks. These results highlight the importance of incorporating conformational variability in MD-based molecular representations.
- *DEMR-HCS: Model Sensitivity to Conformational Regions.* To further understand how models utilize conformational information, we analyzed the learned weights assigned to DEMR features from different RMSD regions. Table 4 reports the average weights across tasks. The results indicate that both models tend to assign higher importance to HLC and MLC conformations, suggesting that structurally diverse molecular states contribute more informative signals for MPP prediction. In contrast, LLC conformations receive relatively low normalized weights than HLC or MLC, further confirming their limited discriminative value. It should be noted that the negative values are caused by the unconstrained linear fusion mechanism. Unlike softmax attention weights, these learned RMSD-region coefficients are not restricted to be non-negative. Positive coefficients indicate that the corresponding region contributes positively to the final representation, whereas negative coefficients suggest suppressive or corrective modulation. In other words, during representation fusion, the model may learn to down-weight or compensate for features from regions assigned negative coefficients.

**Table 4:** Investigation of assigned weights for the frames in different RMSD regions (HLC, MLC and LLC) when learning the DEMR-HCS representations.

Task Category	Feature	Model	Learned Weights		
			HLC	MLC	LLC
TOX21-MD	DEMR-HCS	Transformer	**0.100**	0.053	-0.207
		CNNTransformer	-0.428	**0.258**	-0.123
DUD-E-MD	DEMR-HCS	Transformer	**0.108**	0.057	-0.212
		CNNTransformer	-0.425	**0.257**	-0.123

#### 4.3.3 Time cost analysis.

We further evaluated the computational efficiency of DEMR-based models. Experimental results show that competitive predictive performance can be achieved using only a small number of sampled frames (e.g., 10 frames) in nearly half of the evaluated tasks (NR-AhR, NR-Aromatase, NR-PPAR-gamma, SR-ATAD, SR-MMP, ADA17, and EGFR tasks). This finding suggests that the DEMR representation is capable of retaining essential temporal-spatial patterns even with sparsely sampled frames, thereby achieving a favorable trade-off between computational efficiency and predictive performance. The total training time for such a configuration was approximately 2,000 *s* on an NVIDIA RTX 2080Ti GPU. Given the rapid advancement of GPU architectures and processor technologies, such computational costs are considered acceptable for handling large-scale molecular datasets in MPP studies. Moreover, using more advanced GPUs in the future will reduce such costs further and support dynamics-involved studies in MPP tasks even more.

### 4.4 Discussions

#### 4.4.1 Molecular conformational space analysis.

**Fig 14:**
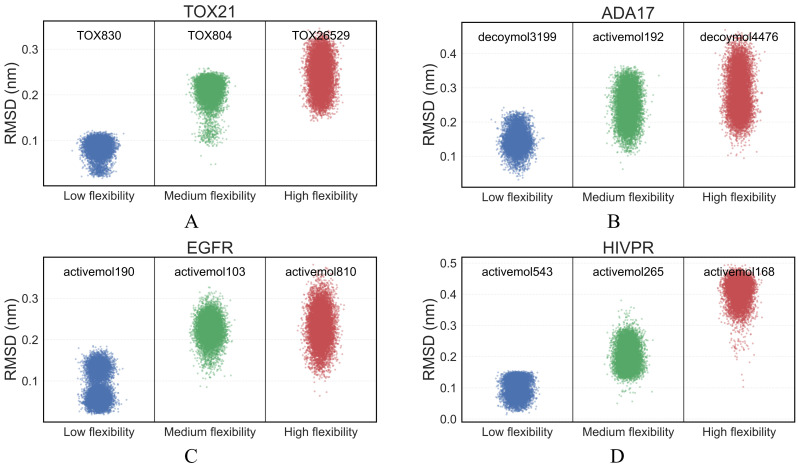
Structural RMSD values during molecular dynamics for molecules with different levels of flexibility. **A.** Representative molecules in TOX21-MD set. **B.** Representative molecules in DUD-E-MD set (ADA17 task). **C.** Representative molecules in DUD-E-MD set (EGFR task). **D.** Representative molecules in DUD-E-MD set (HIVPR task).

We analyzed the molecular flexibility and conformational space. In our study, the number of rotatable bonds (*NRotB*) was uesd to characterize molecular flexibility and molecules were classified into three categories [51]: low-flexibility (NRotB≤7), medium-flexibility (7<NRotB≤10), and high-flexibility (*NRotB* > 10). For molecules with different levels of flexibility, we analyzed the RMSD values between the first structural frame and the subsequent frames in 10 ns MD simulations (sampling frequency: *1ps*). As shown in Fig 14, molecules with different flexibility levels exhibit distinct RMSD distribution patterns. Low-flexibility molecules generally show smaller RMSD fluctuations, indicating that their overall conformations remain close to the initial structure during the simulation. In contrast, high-flexibility molecules generally span a wider range of RMSD values, suggesting that the MD simulations capture larger structural movements for these flexible molecules. This result is consistent with the general understanding that molecules with a larger number of rotatable bonds tend to have greater conformational freedom.

**Fig 15:**
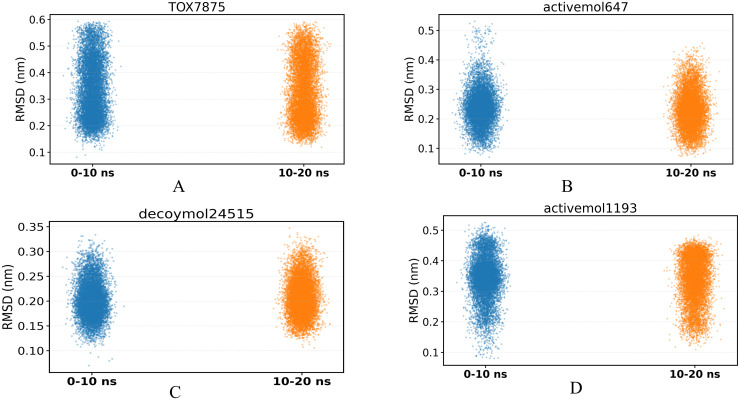
Structural RMSD values from two 10 ns windows of molecular dynamics simulations for high-flexibility molecules. Representative molecules are shown in **A.** TOX21-MD set, **B.** DUD-E-MD set (ADA17 task), **C.** DUD-E-MD set (EGFR task), and **D.** DUD-E-MD set (HIVPR task).

To evaluate whether the 10 ns trajectory is sufficient to describe the major conformational changes of highly flexible molecules, we extended the MD simulations for high-flexibility molecules by an additional 10 ns (for a total of 20 ns) and compared the RMSD plots for the first 10ns with those for the subsequent 10ns. As shown in Fig 15, the RMSD values in the two time windows show no substantial difference, and no obvious systematic increase or distributional shift is observed during the last 10 ns. These results indicate that, under the simulation conditions used in this study, 10 ns of simulation is sufficient to capture the major conformational dynamics of these flexible molecules, and that extending the simulation time provides limited additional information. Overall, although highly flexible molecules exhibit larger RMSD fluctuations, the RMSD analysis shows that the major dynamic conformational changes are already reflected within the first 10 ns. Therefore, for the dynamics-enhanced MPP task investigated in this study, we believe that 10 ns MD simulations are adequate for molecules with different levels of flexibility (which also generally reflects their size, as larger molecules tend to be more flexible).

#### 4.4.2 Analysis of scaffold similarity between the training and test sets.

**Fig 16:**
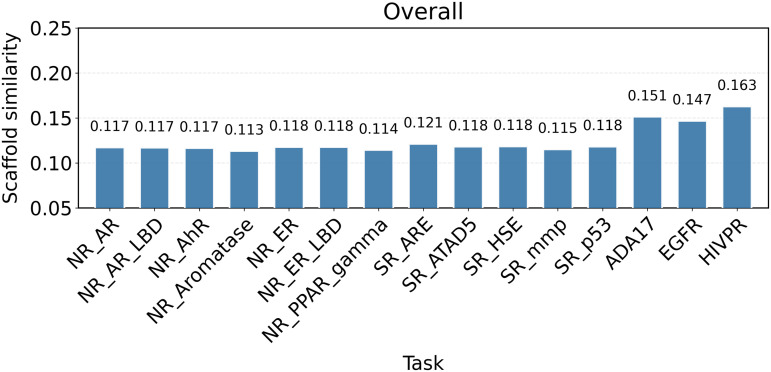
Average scaffold similarity between molecules in the training and test sets for different tasks.

In our study, we used the random splitting strategy to divide the dataset into training, validation, and test sets at a ratio of 7:1:2. The random splitting is a commonly adopted strategy in machine learning. We analyzed the scaffold similarity between the training and test sets. In our study, scaffold similarity was defined as the average *Tanimoto similarity* of the *Morgan fingerprints* computed on the Bemis-Murcko scaffolds of molecule pairs between the training and test sets. Fig 16 reports the average results. As shown in the figure, the average scaffold similarity for each task is low, suggesting that there is no substantial structural similarity leakage between the training and test sets (i.e., scaffold overlap is not significant). In addition, the low average similarity for each task implies that the performance improvements are related to learning the underlying dynamics rather than memorizing similar molecules.

#### 4.4.3 Analysis of statistical significance testing and confidence intervals.

We calculated the 95% confidence intervals using the Student’s *t*-distribution and reported the corresponding interval widths. The results are presented in Table 5. The interval width reflects the uncertainty of the estimated performance across independent runs, where a narrower interval indicates smaller variation and more stable model behavior. The results show that our models have relatively narrow confidence intervals, indicating lower performance variability across runs and suggesting improved stability and robustness. In addition, we compared our two best-performing Transformer models (DEMR-TIS-Transformer and DEMR-RBS-Transformer) against the strongest baseline model (MACCS-LR), as shown in Table 6. The results indicate that our models achieve statistically significant and consistent improvements over the baseline, further supporting the effectiveness of the proposed method.

**Table 5:** Widths of the 95% confidence intervals for model performance.

Feature	Model	TOX21-MD			DUD-E-MD		
		ROC-AUC	Balanced Acc	MCC	ROC-AUC	Balanced Acc	MCC
ECFP	DT	0.308	0.323	0.611	0.129	0.139	0.253
	KNN	0.308	0.273	0.894	0.432	0.204	0.114
	LR	0.363	0.258	0.507	0.005	0.025	0.035
	NB	0.253	0.253	0.378	0.268	0.268	0.253
MACCS	DT	0.278	0.298	0.566	0.134	0.134	0.293
	KNN	0.348	0.363	0.840	0.070	0.109	0.243
	LR	0.318	0.343	0.840	0.015	0.070	0.169
	NB	0.328	0.214	0.318	0.472	0.363	0.691
ECFP	DeepTox	0.293	0.363	0.775	0.010	0.040	**0.065**
Graph	APPNP	0.253	**0.030**	**0.134**	0.114	0.243	0.422
	GAT	0.273	0.159	0.537	0.030	0.164	0.273
	GCN	0.293	0.164	0.542	0.035	0.134	0.243
	GIN	0.378	0.348	0.939	0.025	0.104	0.194
	GraphSAGE	0.283	0.234	0.666	0.045	0.164	0.293
Sequence	ChemBERTa	0.293	0.407	0.686	0.099	0.234	0.388
3D coordinates	EGNN	0.268	0.477	0.790	0.030	0.134	0.179
	CNN	0.224	0.229	0.547	0.015	0.084	0.169
DEMR-TIS	Transformer	**0.084**	0.099	0.199	**0.005**	**0.030**	**0.065**
	CNNTransformer	**0.159**	0.258	0.139	**0.010**	0.055	0.159
DEMR-RBS	Transformer	**0.045**	0.089	0.174	**0.005**	0.040	0.119
	CNNTransformer	**0.184**	0.263	0.199	0.020	0.050	0.119
DEMR-HCS	Transformer	**0.045**	0.104	0.179	0.015	0.089	0.234
	CNNTransformer	**0.199**	0.378	0.194	0.035	0.055	0.134

**Table 6:** Statistical significance test against the LR baseline.

Method	Wins/Tasks	Wilcoxon *p*	Holm-adjusted *p*
DEMR-TIS-Transformer	12/15	0.0021	0.0128^*^
DEMR-RBS-Transformer	12/15	0.0027	0.0134^*^

#### 4.4.4 Performance analysis across different molecular flexibility levels.

We designed a flexibility-aware adaptive sampling (DEMR-FAS) strategy to construct DEMR sequence inputs for molecules with different flexibility levels. Specifically, low-, medium-, and high-flexibility molecules were sampled with different frequencies (50, 20, or 10) respectively, allowing more flexible molecules to retain more trajectory frames and capture richer conformational variations. As a result, we used sequence lengths of 200, 500, and 1000 for molecules with different flexibility levels. During model training, the padding-mask strategy was used to handle DEMR sequences with different lengths. The results are shown in Table 7. This sampling strategy achieves comparable performance on the CNNTransformer model, and it suggests that adaptively selecting input sequence lengths according to molecular or task-specific characteristics remains a worthwhile direction for future exploration.

**Table 7:** Performance of our models using the flexibility-aware adaptive sampling strategy.

Feature	Model	TOX21-MD			DUD-E-MD		
		ROC-AUC	Balanced Acc	MCC	ROC-AUC	Balanced Acc	MCC
DEMR-FAS	Transformer	0.735±0.122	0.705±0.176	0.270±0.146	0.964±0.080	0.910±0.098	0.824±0.237
	CNNTransformer	0.717±0.077	0.565±0.098	0.269±0.122	0.924±0.098	0.850±0.154	0.839±0.094

#### 4.4.5 Sequence length sensitivity analysis.

**Fig 17:**
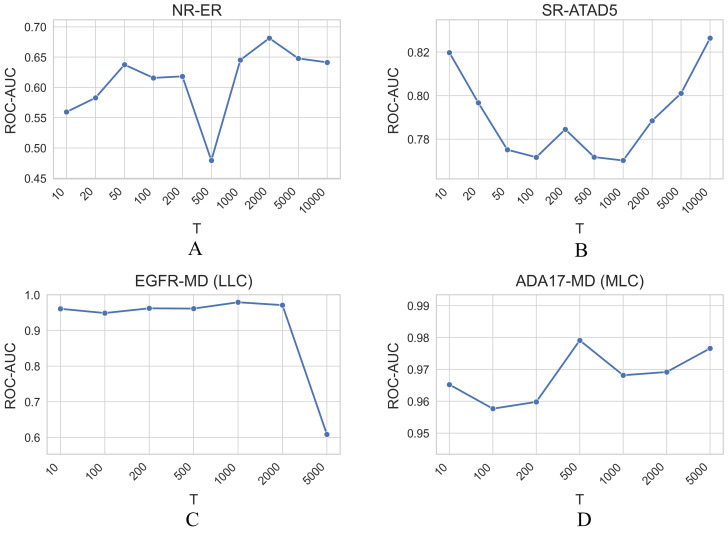
Sensitivity of predictive performance of our models to the sequence length parameter *T.* Representative models, including **A.** DEMR-TIS-CNNTransformer, **B.** DEMR-TIS-Transformer, **C.** DEMR-RBS-CNNTransformer (LLC), and **D.** DEMR-RBS-Transformer (MLC), were investigated.

We examined how the model’s ROC-AUC changes as the sequence length *T* increases for different tasks. Although there is no unified pattern across models or tasks, several interesting trends were found. Fig 17 illustrates some representative examples. ① DEMR-TIS-CNNTransformer on NR-ER task in Fig 17A. Increasing the sequence length generally preserves more dynamics information and leads to better performance, but there is a performance valley around *T* = 500. ② DEMR-TIS-Transformer on SR-ATAD5 task in Fig 17B. Shorter and longer sequence lengths yield better performance, but there is a performance valley for intermediate lengths (between *T* = 50 and *T* = 1000). ③ DEMR-RBS-CNNTransformer (LLC) on EGFR task in Fig 17C. Shorter and intermediate sequence lengths result in similar performance, but there is a severe performance drop at the longest sequence length (*T* = 5000). ④ DEMR-RBS-Transformer (MLC) on ADA17 task in Fig 17D. Increasing the sequence length gradually enhances the model performance. Overall, increasing *T* can preserve more dynamic or conformational information, but its benefits do not increase monotonically with *T*. A smaller *T* may be insufficient to represent molecular dynamics, whereas an excessively large *T* may lead to information redundancy, increase computational cost, and cause training instability. Deeper insights into these relationship patterns will be pursued in our future work.

#### 4.4.6 Analysis for the learned positional encoding.

**Fig 18:**
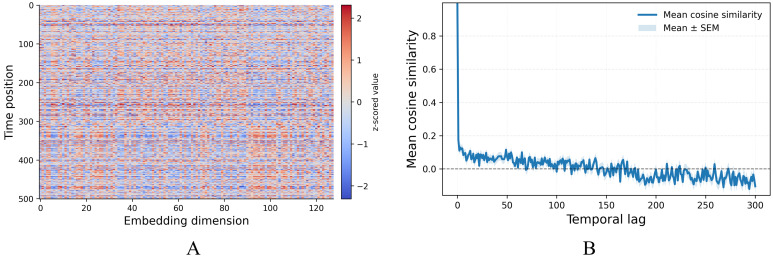
The learned positional encoding of a representative molecule (ID: activemol1187) in DUD-E-MD set (HIVPR task) from DEMR-TIS-CNNTransformer (*Freq* = 20). **A.** Time-varying positional embeddings of the molecule. **B.** Average cosine similarity at different temporal lags.

We also investigated the learned positional encoding of molecular trajectories from DEMR-TIS-CNNTransformer. We observed interesting patterns in some molecules, exemplified in Fig 18 for the molecule with ID *activemol1187* from DUD-E-MD set (HIVPR task). Fig 18A shows the positional embeddings learned by our model for this molecule. Based on these embeddings, we computed the average cosine similarity at different temporal lags, as shown in Fig 18B. The similarity rapidly decreases with increasing lag and gradually approaches 0, with slightly negative values at larger lags. This indicates that frames farther apart in time become progressively decorrelated, reflecting a temporal structure similar to a decaying memory kernel. A more comprehensive characterization of these patterns across molecules will be pursued in future work to derive deeper scientific insights.

## 5 Conclusions

In this paper, we conducted comprehensive MD simulations on several representative MPP datasets and established a series of large-scale molecular dynamics datasets specifically designed for MPP tasks. These curated datasets occupy approximately 200 GB of storage and are publicly available, providing a valuable benchmark resource for future dynamics-driven molecular modeling research. This contribution not only enriches the available data foundation for MPP studies but also opens up new possibilities for exploring temporal and conformational information in molecular representations. Based on these dynamics data, we further proposed the molecular representation, DEMR, and two deep learning methods for dynamics-based MPP tasks. Extensive experiments and analyses demonstrated that our approach achieved better performance for more challenging MPP tasks. Moreover, the experimental results showed that a properly configured sampling frequency can effectively enhance the representation ability of DEMR and greatly improve the model performance in MPP tasks. On the other hand, DEMR located in the HLC and MLC regions are often more advantageous in guiding an accurate MPP model.

## Supporting information

S1 Data This file contains detailed model performance metrics and results. (XLSX)

S2 Data This file contains the supplementary data used to generate the figures in the main text. (XLSX)
