# Supplementary material for: Dynamics-enhanced molecular property prediction guided by deep learning
Source: PLoS Comput Biol. 2026 Jul 16;22(7):e1014515. doi: 10.1371/journal.pcbi.1014515 (PMC13390958; doi:10.1371/journal.pcbi.1014515)
Supplement: Attachment — Submitted filename: ResponseLetter.pdf [file pcbi.1014515.s004.pdf]

Response Letter

Manuscript ID:PCOMPBIOL-D-26-00323
Manuscript Title: Dynamics-enhanced Molecular Property Prediction Guided by Deep Learning

Author List: Qiang Liu, Debby D. Wang, Weiqing Guo, Yuting Huang, Xizhao Wang

June 26, 2026

Dear Editor:

We would like to thank the editor and reviewers for their helpful comments, and we have revised the manuscript according to their suggestions. Point-by-point responses are enclosed, and the changes are highlighted in blue color in the revised manuscript. With the submission of this manuscript, we would like to undertake the responsibility that:

(i) this manuscript is the authors' original work, which has not been published nor submitted simultaneously elsewhere;

(ii) all authors have checked the revised manuscript and agreed to this submission;

(iii) there is no conflict of interest.

Thank you very much for your time and effort in handling our manuscript, and we look forward to receiving further responses from your office.

Yours Sincerely,

Qiang Liu

1 Response to Reviewer #1

Thank you for your valuable comments, which have provided important guidance for revising our paper as follows:

Comment 1. *Transferability to out-of-distribution new molecules is one of the essential tasks for these models. The authors have shown in Fig. 2 the similarities of molecules within tasks, separated into positive, negative, and inter-class. Can the authors show a similar figure comparing the training and test sets? Also, can you describe how this similarity is defined? If they are fingerprint-based, I am quite surprised that the DUD-E set similarities are this low.*

Authors' Response: In the revised manuscript, we have added a new figure (Figure 8) that shows the similarities between molecules in the training set and those in the test set. It can be seen that the average similarity between molecules in the two sets is also low.

Here, similarity is defined as the average *Tanimoto similarity* of the *Morgan fingerprints* for molecule pairs within a single set (e.g., positive or negative) or between two sets (e.g., positive vs. negative, or training vs. test). Specifically, the *Morgan fingerprint* of each molecule was generated using the `RDKit` package from its *SMILES* representation, with default parameters (radius = 2, length = 2048). *Tanimoto* on *Morgan fingerprints* quantifies how similar two molecular structures are in terms of their substructures. These details have been clarified in the revised manuscript (highlighted in Pages 5 and 10).

For the *DUD-E* database, we selected several well-studied therapeutic targets (e.g., `ADA17`, `EGFR`, and `HIVPR`) and their corresponding ligands (actives and decoys) for target-specific predictions. According to our calculations, both the intra-class and inter-class pairwise molecular similarities are low, indicating that the molecules are diverse and representative.

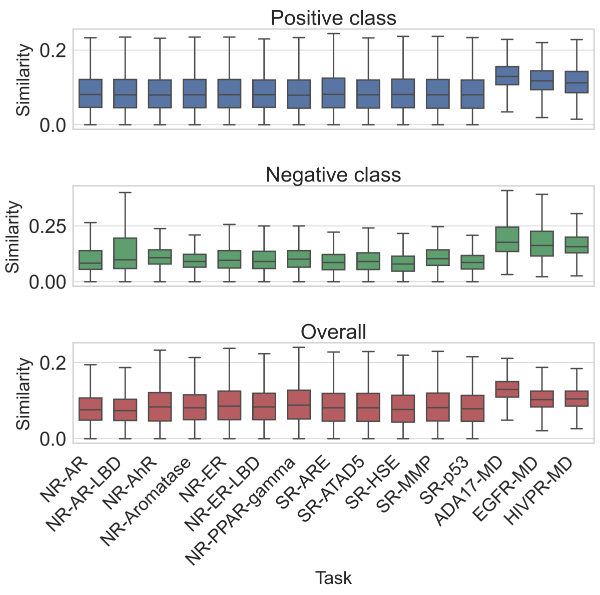

Figure 8: Pairwise similarity of molecules between the training and test sets.

Comment 2. *It would be interesting to see what the learned positional encoding looks like. It would significantly enhance this paper if you can show if it looks similar to a memory kernel.*

Authors' Response: In the revised manuscript, we have added a discussion (highlighted in Pages 20 and 21) of the learned positional encoding of molecular trajectories from `DEMR-TIS`-CNNTransformer. We observed interesting patterns in some molecules, exemplified in Figure 18 for the molecule with ID *activemol1187* from DUD-E-MD set (HIVPR task). Figure 18A shows the positional embeddings learned by our model for this molecule. Based on these embeddings, we computed the average cosine similarity at different temporal lags, as shown in Figure 18B. The similarity rapidly decreases with increasing lag and gradually approaches 0, with slightly negative values at larger lags. This indicates that frames farther apart in time become progressively decorrelated, reflecting a temporal structure similar to a decaying memory kernel. A more comprehensive characterization of these patterns across molecules will be pursued in future work to derive deeper scientific insights.

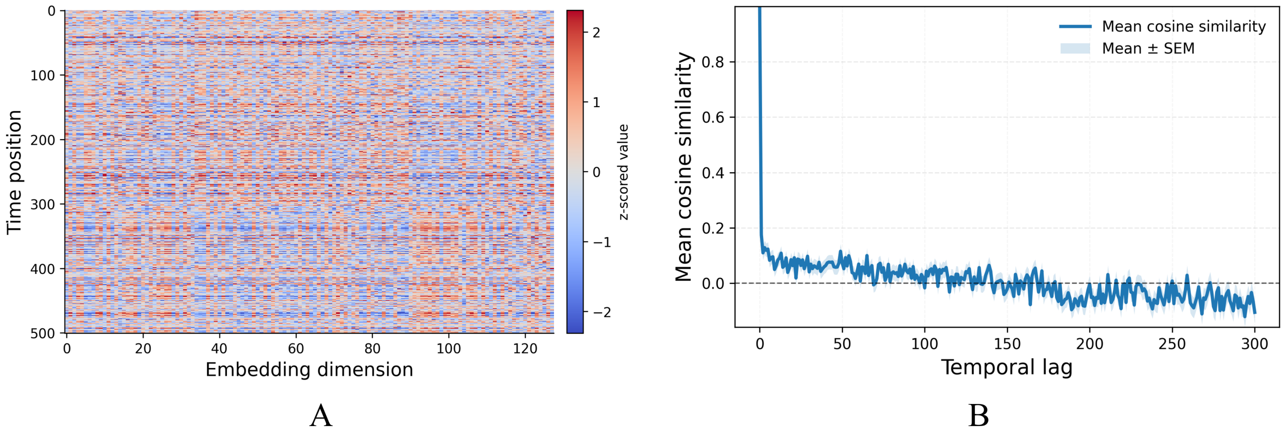

Figure 18: The learned positional encoding of a representative molecule (ID: activemol1187) in DUD-E-MD set (HIVPR task) from `DEMR-TIS`-CNNTransformer ($Freq = 20$). A. Time-varying positional embeddings of the molecule. B. Average cosine similarity at different temporal lags.

Comment 3. *A presentation issue: In almost every figure (maybe except Fig. 4), the text is too small and very hard to read. Can you set your Matplotlib DPI to 300 and adjust the font sizes? Also, in the manuscript, you refer to the three sampling strategies as circled 1, 2, and 3. This circled number notation is used elsewhere (e.g., lines 303-313), which can create a minor clarity issue. You have given your three strategies very good names (Section 3.2, Lines 206-215); I suggest using these names throughout the manuscript.*

Authors' Response: In the revised manuscript, we have increased the resolution (DPI = 300) and adjusted the font sizes of all figures. Moreover, we have renamed the three sampling strategies to `DEMR-TIS`, `DEMR-RBS`, and `DEMR-HCS` in the revised manuscript to avoid potential confusion.

2 Response to Reviewer #2

We appreciate the constructive feedback on our paper. Below is a point-by-point response to your comments.

Comment 1. *The novelty term is overstated ("fist application..."), MD-informed representations and ensemble-based conformer learning have appeared in binding affinity prediction and 3D-GNN literature, I believe even cited by the Authors. The manuscript should:*

- *Carefully position itself relative to equivariant 3D GNNs and ensemble conformer learning.*
- *Clarify what is genuinely novel: the specific atom-pair-distance tensor? the systematic MD dataset? the RMSD-weighting strategy?*

Authors' Response:

In the revised manuscript, we removed the term "first application" to avoid overstating the novelty.

- ▶ We further clarified the differences between EGNNs, ensemble conformational learning methods, and our proposed method. EGNNs focus on 3D molecular geometry by considering a single conformation or a set of conformations, while ensemble conformational learning methods account for the coexistence of multiple molecular conformations. However, the resulting molecular representations are still essentially static and independent, lacking both temporal ordering and dynamic evolution information (highlighted in Pages 3 and 4 of the revised manuscript). To fill this gap, our models leverage temporal dependencies in MD trajectories for MPP tasks, with the potential to further improve prediction performance.

- ▶ We clarified the novelty of this work, including ① constructing systematic MD datasets for MPP tasks; ② leveraging MD information for MPP within deep learning frameworks; and ③ investigating conformation sampling strategies for dynamics-involved MPP tasks. These points are highlighted in page 3 of the revised manuscript.

Comment 2. *For DUD-E tasks, reported ROC-AUC values near (or exactly) 1.00 (Table 3, p. 11) are suspiciously high. DUD-E is known to allow artificial separation between actives and decoys.*

The authors should clarify:

- *Was scaffold split used?*
- *Were conformers from the same molecule ever distributed across train/test?*
- *Was any chemical similarity control performed?*
- *Without scaffold-level splitting, improvements may reflect memorization rather than dynamics learning.*

Authors' Response: In the revised manuscript, we have provided an explanation regarding these issues. Specifically,

- ▶ We used a random split, which is widely adopted in machine learning research, for our experiments. To further investigate scaffold distributions between the training and test sets, we have added a discussion (highlighted in Pages 17 and 18) of scaffold similarity between molecules in the training and testing sets. Here, scaffold similarity is defined as the average *Tanimoto similarity* of the *Morgan fingerprints* computed on the `Bemis-Murcko scaffolds` of molecule pairs between the training and test sets. We found that the average scaffold similarity for each task is low (Figure 16).

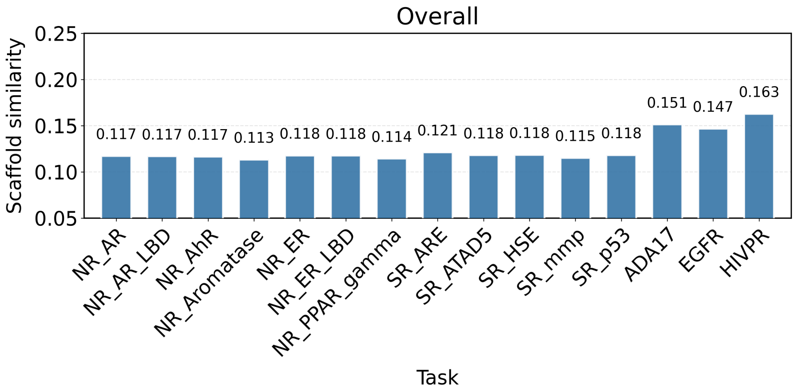

Figure 16: Average scaffold similarity between molecules in the training and test sets for different tasks.

- ▶ We split the data into training and testing sets by molecule, meaning that all conformers of the same molecule are assigned to the same set (either training or test). We have clarified this in the revised manuscript (highlighted in Page 10).

- ▶ Prior to predictive modeling, we calculated the molecular similarity between the training and test sets as a similarity control. Specifically, similarity is defined as the average *Tanimoto similarity* of the *Morgan fingerprints* for molecule pairs between the training and test sets. As shown in Fig. 8, the average similarity between the training and test sets is low, whether considering the positive class, the negative class, or all samples (highlighted in Page 11).

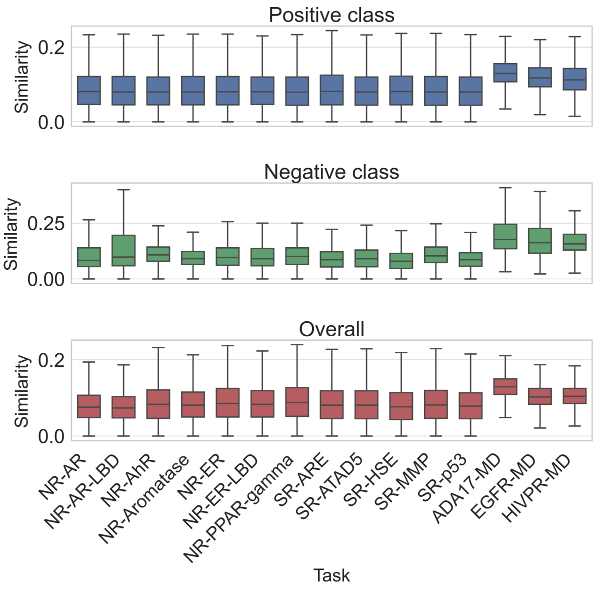

Figure 8: Pairwise similarity of molecules between the training and test sets.

- ▶ Although a scaffold split was not used in this study, we assessed scaffold similarity between molecules in the training and test sets obtained with the random split strategy as a scaffold-level similarity control (Figure 16 as above). The low average similarity for each task implies that the performance improvements are related to learning the underlying dynamics rather than memorizing similar molecules.

Comment 3. *Each molecule is simulated for 10 ns. For many flexible ligands, 10 ns may not sufficiently sample relevant conformational space. Could you discuss this and provide the explanation why 10 ns is sufficient for all molecule sizes (4–197 atoms)?*

Authors' Response: Thank you for this constructive suggestion. In the revised manuscript, we have added discussions of molecular flexibility and conformational space.

First, following *Veberet al.* [51], we defined molecular flexibility using the *number of rotatable bonds* ($NRotB$) and classified molecules into the following three types.

- $NRotB \leq 7$: low-flexibility molecule.
- $7 < NRotB \leq 10$: medium-flexibility molecule.
- $NRotB > 10$: high-flexibility molecule.

Second, we conducted two studies to assess whether MD simulations of 10ns are enough to model the conformational space of high-flexibility molecules. ① For molecules with different levels of flexibility, we analyzed the RMSD values between the first structural frame and the subsequent frames in 10ns MD simulations (sampling frequency: *1ps*). As shown in the RMSD plots (*Figure* 14), high-flexibility molecules generally span a wider range of RMSD values, suggesting that the MD simulations capture larger structural movements for these flexible molecules. ② For high-flexibility molecules, we extended the MD simulations by an additional 10ns (for a total of 20ns) and compared the RMSD plots for the first 10ns with those for the subsequent 10ns. As shown in the RMSD plots (*Figure* 15), the RMSD values in the two time windows do not differ much. This suggests that 10ns of simulation is sufficient to capture the major conformational dynamics of these flexible molecules, and that extending the simulation time provides limited additional information.

Overall, we believe that 10 ns MD simulations are adequate for molecules with different levels of flexibility (which also generally reflects their size, as larger molecules tend to be more flexible). These points are now discussed in the *Discussions Section*(highlighted in Pages 15 and 17) of the revised manuscript.

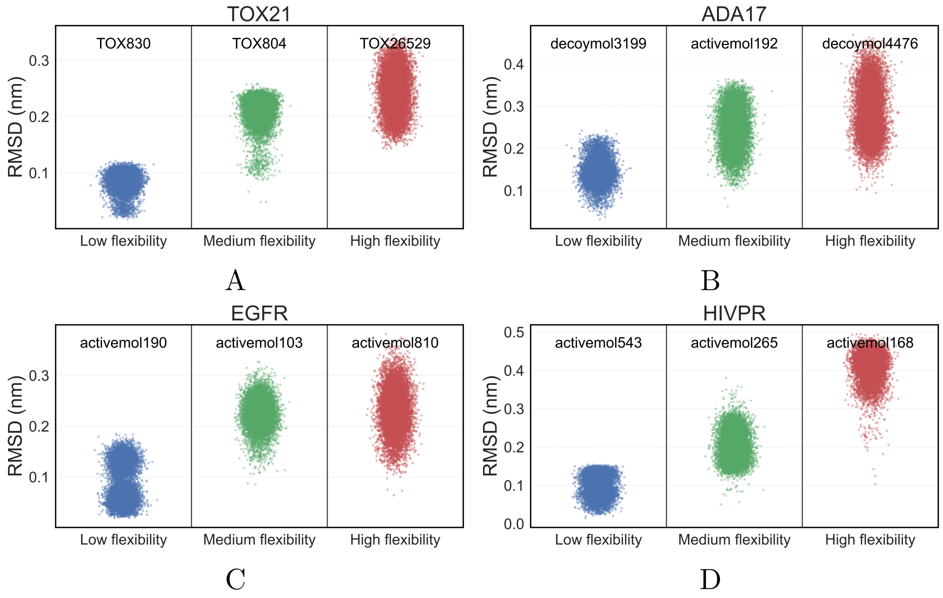

Figure 14: Structural RMSD values during molecular dynamics for molecules with different levels of flexibility. A. Representative molecules in TOX21-MD set. B. Representative molecules in DUD-E-MD set (ADA17 task). C. Representative molecules in DUD-E-MD set (EGFR task). D. Representative molecules in DUD-E-MD set (HIVPR task).

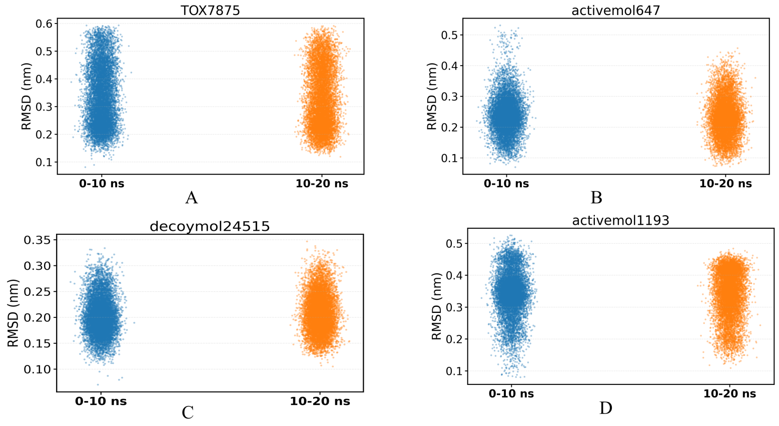

Figure 15: Structural RMSD values from two 10 ns windows of molecular dynamics simulations for high-flexibility molecules. Representative molecules are shown in A. TOX21-MD set, B. DUD-E-MD set (ADA17 task), C. DUD-E-MD set (EGFR task), and D. DUD-E-MD set (HIVPR task).

References:

[51] Veber, D. F., Johnson, S. R., Cheng, H. Y., Smith, B. R., Ward, K. W., & Kopple, K. D. "Molecular properties that influence the oral bioavailability of drug candidates". Journal of medicinal chemistry, 45(12), 2615-2623 (2002).

Comment 4. *DEMR encodes: Atom-type pairs (8×8) and Distance bins (3 ranges). This is effectively a histogram-based 3D descriptor. Please provide explanation:*

- *How does this compare to modern equivariant GNNs using full 3D coordinates?*
- *Is the improvement due to dynamics or simply due to using 3D information?*
- *Is there a baseline using single static 3D conformer with the same encoding? What about 3D static control as the evaluation point?*

Authors' Response:

In the revised manuscript, we have strengthened the experimental section, and the results are now reported in Table 3.

- We added a baseline using modern equivariant graph neural networks (EGNNs) with full 3D atomic coordinates. The 3D atomic coordinates of the molecules in their initial MD simulation states, optimized with *MMFF*/*UFF*, were fed into the EGNNs for predictive modeling. As shown in Table 3, this baseline often underperforms our `DEMR-based Transformers`. More details can be found in Pages 12 and 13 of the revised manuscript.

- Based on the performance of this baseline, we believe that the performance improvements are primarily due to modeling molecular dynamics rather than using 3D structural information. It validates the importance of conformational dynamics for molecular property prediction.

- Moreover, we added another baseline using convolutional neural networks (CNNs) with single static 3D molecular conformations. The 3D atomic coordinates of the molecules in their initial MD simulation states were fed into the CNNs for predictive modeling. Also, this baseline underperforms our `DEMR-based Transformers`, as shown in Table 3. More details can be found in Page 13 of the revised manuscript. This again suggests that the performance improvements are driven by dynamics rather than by the use of static 3D molecular information.

Table 3: Model performance comparison for MPP tasks.

Feature	Model	TOX21-MD			DUD-E-MD		
		ROC-AUC	Balanced Acc	MCC	ROC-AUC	Balanced Acc	MCC
ECFP	DT	0.631 ± 0.062	0.625 ± 0.065	0.257 ± 0.123	0.969 ± 0.026	0.969 ± 0.028	0.937 ± 0.051
	KNN	0.679 ± 0.062	0.554 ± 0.055	0.199 ± 0.180	0.868 ± 0.087	0.634 ± 0.041	0.352 ± 0.023
	LR	0.732 ± 0.073	0.628 ± 0.052	0.285 ± 0.102	**0.998** ± 0.001	0.989 ± 0.005	0.982 ± 0.007
	NB	0.564 ± 0.051	0.564 ± 0.051	0.094 ± 0.076	0.805 ± 0.054	0.805 ± 0.054	0.678 ± 0.051
MACCS	DT	0.647 ± 0.056	0.632 ± 0.060	0.253 ± 0.114	0.947 ± 0.027	0.947 ± 0.027	0.891 ± 0.059
	KNN	0.734 ± 0.070	0.605 ± 0.073	0.304 ± 0.169	0.984 ± 0.014	0.947 ± 0.022	0.889 ± 0.049
	LR	0.764 ± 0.064	0.603 ± 0.069	0.284 ± 0.169	0.996 ± 0.003	0.981 ± 0.014	0.954 ± 0.034
	NB	0.564 ± 0.066	0.529 ± 0.043	0.046 ± 0.064	0.887 ± 0.095	0.854 ± 0.073	0.704 ± 0.139
ECFP	DeepTox	0.767 ± 0.059	0.627 ± 0.073	**0.359** ± 0.156	**0.998** ± 0.002	**0.992** ± 0.008	**0.985** ± 0.013
Graph	APPNP	0.711 ± 0.051	0.502 ± 0.006	0.009 ± 0.027	0.921 ± 0.023	0.799 ± 0.049	0.626 ± 0.085
	GAT	0.731 ± 0.055	0.516 ± 0.032	0.062 ± 0.108	0.992 ± 0.006	0.950 ± 0.033	0.914 ± 0.055
	GCN	0.730 ± 0.059	0.516 ± 0.033	0.060 ± 0.109	0.982 ± 0.007	0.941 ± 0.027	0.891 ± 0.049
	GIN	0.756 ± 0.076	0.559 ± 0.070	0.187 ± 0.189	0.989 ± 0.005	0.967 ± 0.021	0.938 ± 0.039
	GraphSAGE	0.748 ± 0.057	0.527 ± 0.047	0.101 ± 0.134	0.988 ± 0.009	0.943 ± 0.033	0.900 ± 0.059
Sequence	ChemBERTa	0.736 ± 0.059	0.553 ± 0.082	0.206 ± 0.138	0.974 ± 0.020	0.917 ± 0.047	0.845 ± 0.078
3D coordinates	EGNN	0.748 ± 0.054	0.576 ± 0.096	0.193 ± 0.159	0.991 ± 0.006	0.955 ± 0.027	0.823 ± 0.036
	CNN	0.678 ± 0.045	0.604 ± 0.046	0.307 ± 0.110	0.987 ± 0.003	0.948 ± 0.017	0.889 ± 0.034
DEMR-TIS	Transformer	**0.813** ± 0.017	**0.73** ± 0.020	0.339 ± 0.040	0.995 ± 0.001	0.967 ± 0.006	0.925 ± 0.013
	CNNTransformer	0.760 ± 0.032	**0.670** ± 0.052	0.290 ± 0.028	0.989 ± 0.002	0.949 ± 0.011	0.885 ± 0.032
DEMR-RBS	Transformer	**0.811** ± 0.009	**0.726** ± 0.018	0.330 ± 0.035	0.993 ± 0.001	0.958 ± 0.008	0.901 ± 0.024
	CNNTransformer	0.702 ± 0.037	0.616 ± 0.053	0.192 ± 0.040	0.982 ± 0.004	0.938 ± 0.010	0.857 ± 0.024
DEMR-HCS	Transformer	**0.809** ± 0.009	**0.726** ± 0.021	0.317 ± 0.036	0.993 ± 0.003	0.958 ± 0.018	0.899 ± 0.047
	CNNTransformer	0.708 ± 0.040	0.611 ± 0.076	0.209 ± 0.039	0.983 ± 0.007	0.962 ± 0.011	0.884 ± 0.027

Comment 5.*Some of the results are reported as single averages. Given performance differences of 0.03–0.05 ROC-AUC there are missing:*

- *Standard deviations across runs.*
- *Statistical significance testing.*
- *Confidence intervals.*

Authors' Response: In the revised manuscript, we have conducted multiple runs using different random seeds. Specifically,

- For each task, experiments were repeated three times with different random seeds, and the standard deviation across runs are now reported in Table 3 as above (Page 13 of the revised manuscript).
- We calculated 95% confidence intervals using Student's t-distribution and reported the corresponding interval widths. The results are now presented in Table 5 and show that our models have relatively narrow confidence intervals, indicating lower performance variability across runs and improved stability and robustness (Pages 18 and 19 of the revised manuscript).

Table 5: Widths of the 95% confidence intervals for model performance.

Feature	Model	TOX21-MD			DUD-E-MD		
		ROC-AUC	Balanced Acc	MCC	ROC-AUC	Balanced Acc	MCC
ECFP	DT	0.308	0.323	0.611	0.129	0.139	0.253
	KNN	0.308	0.273	0.894	0.432	0.204	0.114
	LR	0.363	0.258	0.507	0.005	0.025	0.035
	NB	0.253	0.253	0.378	0.268	0.268	0.253
MACCS	DT	0.278	0.298	0.566	0.134	0.134	0.293
	KNN	0.348	0.363	0.840	0.070	0.109	0.243
	LR	0.318	0.343	0.840	0.015	0.070	0.169
	NB	0.328	0.214	0.318	0.472	0.363	0.691
ECFP	DeepTox	0.293	0.363	0.775	0.010	0.040	**0.065**
Graph	APPNP	0.253	**0.030**	**0.134**	0.114	0.243	0.422
	GAT	0.273	0.159	0.537	0.030	0.164	0.273
	GCN	0.293	0.164	0.542	0.035	0.134	0.243
	GIN	0.378	0.348	0.939	0.025	0.104	0.194
	GraphSAGE	0.283	0.234	0.666	0.045	0.164	0.293
Sequence	ChemBERTa	0.293	0.407	0.686	0.099	0.234	0.388
3D coordinates	EGNN	0.268	0.477	0.790	0.030	0.134	0.179
	CNN	0.224	0.229	0.547	0.015	0.084	0.169
DEMR-TIS	Transformer	**0.084**	0.099	0.199	**0.005**	**0.030**	**0.065**
	CNNTransformer	**0.159**	0.258	0.139	**0.010**	0.055	0.159
DEMR-RBS	Transformer	**0.045**	0.089	0.174	**0.005**	0.040	0.119
	CNNTransformer	**0.184**	0.263	0.199	0.020	0.050	0.119
DEMR-HCS	Transformer	**0.045**	0.104	0.179	0.015	0.089	0.234
	CNNTransformer	**0.199**	0.378	0.194	0.035	0.055	0.134

- As a statistical significance analysis, we tested whether our models perform significantly better than the baseline. Specifically, we compared our two best-performing Transformer models (`DEMR-TIS`-Transformer and `DEMR-RBS`-Transformer) against the strongest baseline model (MACCS-LR), as shown in Table 6. The results indicate that our models achieve statistically significant and consistent improvements over the baseline (Page 19 of the revised manuscript).

Table 6: Statistical significance test against the LR baseline.

Method	Wins/Tasks	Wilcoxon p	Holm-adjusted p
DEMR-TIS- Transformer	12/15	0.0021	0.0128*
DEMR-RBS-Transformer	12/15	0.0027	0.0134*

Comment 6. *Some RMSD-region weights are negative. Interpretation is unclear. Why are HLC weights sometimes negative for CNN Transformer?*

Authors' Response:

The negative values are caused by the unconstrained linear fusion mechanism. Unlike *softmax* attention weights, these learned RMSD-region coefficients are not restricted to be non-negative. The learned values represent contribution coefficients for different RMSD regions, which modulate the DEMR representations corresponding to those conformational regions. Positive coefficients indicate that the corresponding region contributes positively to the final representation, whereas negative coefficients suggest suppressive or corrective modulation. In other words, the model may learn to down-weight or compensate for features from regions assigned negative coefficients during representation fusion (Pages 14 and 15 of the revised manuscript).

Comment 7. *Authors may wish to provide the discussion of sequence length sensitivity when T increases.*

Authors' Response: Thanks for this suggestion. We have discussed the sensitivity of model performance to sequence length in the revised manuscript (highlighted in Pages 19 and 20).

First, we examined how the model's ROC-AUC changes as the sequence length increases for different tasks. Although there is no unified pattern across models or tasks, several interesting trends were found. Figure 17 illustrates some representative examples. ① **`DEMR-TIS`-CNNTransformer on NR-ER task in Fig 17A.** Increasing the sequence length generally preserves more dynamics information and leads to better performance, but there is a performance valley around $T = 500$. ② **`DEMR-TIS`-Transformer on SR-ATAD5 task in Fig 17B.** Shorter and longer sequence lengths yield better performance, but there is a performance valley for intermediate lengths (between $T = 50$ and $T = 1000$). ③ **`DEMR-RBS`-CNNTransformer (LLC) on EGFR task in Fig 17C.** Shorter and intermediate sequence lengths result in similar performance, but there is a severe performance drop at the longest sequence length ($T = 5000$). ④ **`DEMR-RBS`-Transformer (MLC) on ADA17 task in Fig 17D.** Increasing the sequence length gradually enhances the model performance. Deeper insights into these relationship patterns will be pursued in our future work.

Second, we designed a flexibility-aware adaptive sampling (DEMR-FAS) strategy to construct DEMR sequences. Specifically, low-, medium-, and high-flexibility molecules were sampled with different frequency (50, 20, or 10) respectively, allowing more flexible molecules to retain more trajectory frames and capture richer conformational variations. As a result, we used sequence lengths of 200, 500, and 1000 for molecules with different flexibility levels (as defined in Comment 3). A padding-mask strategy was used to handle DEMR sequences with different lengths. The performance of our models based on this sampling stratey is shown in Table 7. This sampling strategy achieves comparable performance on the CNNTransformer model, and it suggests that adaptively selecting input sequence lengths according to molecular or task-specific characteristics remains a worthwhile direction for future exploration.

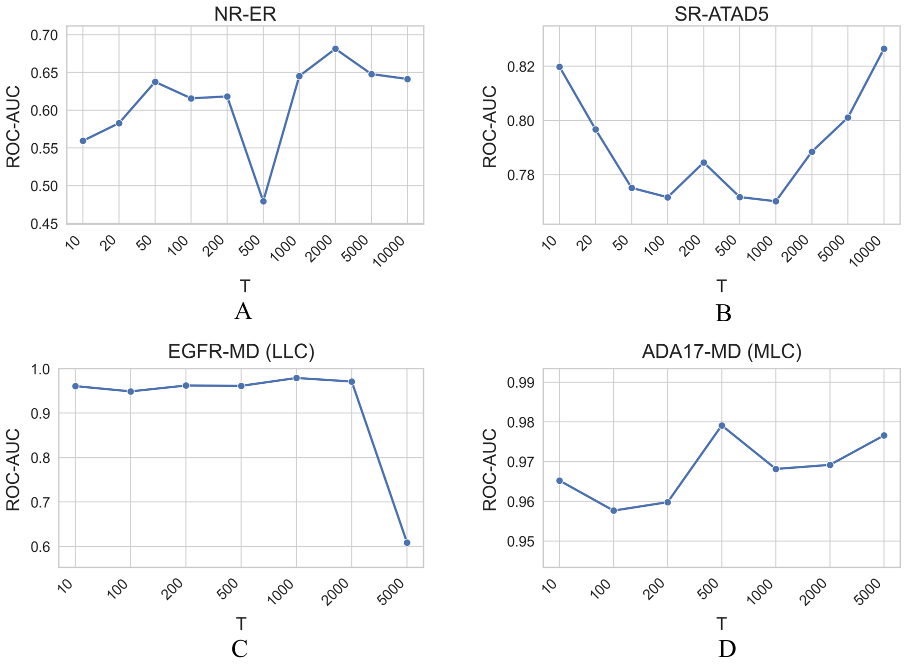

Figure 17: Sensitivity of predictive performance of our models to the sequence length parameter T. Representative models, including A. `DEMR-TIS`-CNNTransformer, B. `DEMR-TIS`-Transformer, C. `DEMR-RBS`-CNNTransformer (LLC), and D. `DEMR-RBS`-Transformer (MLC), were investigated.

Table 7: Performance of our models using the flexibility-aware adaptive sampling strategy.

Feature	Model	TOX21-MD			DUD-E-MD		
		ROC-AUC	Balanced Acc	MCC	ROC-AUC	Balanced Acc	MCC
DEMR-FAS	Transformer	0.735 ± 0.122	0.705 ± 0.176	0.270 ± 0.146	0.964 ± 0.080	0.910 ± 0.098	0.824 ± 0.237
	CNNTransformer	0.717 ± 0.077	0.565 ± 0.098	0.269 ± 0.122	0.924 ± 0.098	0.850 ± 0.154	0.839 ± 0.094
