# Supplementary material for: Dynamics-enhanced molecular property prediction guided by deep learning
Source: PLoS Comput Biol. 2026 Jul 16;22(7):e1014515. doi: 10.1371/journal.pcbi.1014515 (PMC13390958; doi:10.1371/journal.pcbi.1014515)
Supplement: Peer review history

# Peer review: Decision Letter 0

**DOI:** 10.1371/journal.pcbi.1014515.r001

**Authors:** Mark Alber

28 Apr 2026

PCOMPBIOL-D-26-00323

Dynamics-enhanced Molecular Property Prediction Guided by Deep Learning

PLOS Computational Biology

Dear Dr. Wang,

Thank you for submitting your manuscript to PLOS Computational Biology. After careful consideration, we feel that it has merit but does not fully meet PLOS Computational Biology's publication criteria as it currently stands. Therefore, we invite you to submit a revised version of the manuscript that addresses the points raised during the review process.

Please submit your revised manuscript by Jun 28 2026 11:59PM. If you will need more time than this to complete your revisions, please reply to this message or contact the journal office at ploscompbiol@plos.org. When you're ready to submit your revision, log on to https://www.editorialmanager.com/pcompbiol/ and select the 'Submissions Needing Revision' folder to locate your manuscript file.

Please include the following items when submitting your revised manuscript:

* A letter that responds to each point raised by the editor and reviewer(s). You should upload this letter as a separate file labeled 'Response to Reviewers'. This file does not need to include responses to formatting updates and technical items listed in the 'Journal Requirements' section below.

* A marked-up copy of your manuscript that highlights changes made to the original version. You should upload this as a separate file labeled 'Revised Manuscript with Track Changes'.

* An unmarked version of your revised paper without tracked changes. You should upload this as a separate file labeled 'Manuscript'.

If you would like to make changes to your financial disclosure, competing interests statement, or data availability statement, please make these updates within the submission form at the time of resubmission. Guidelines for resubmitting your figure files are available below the reviewer comments at the end of this letter

We look forward to receiving your revised manuscript.

Kind regards,

Mark Alber, Ph.D.

Section Editor

PLOS Computational Biology

Mark Alber

Section Editor

PLOS Computational Biology

**Journal Requirements:**

1) Please ensure that the CRediT author contributions listed for every co-author are completed accurately and in full.

At this stage, the following Authors/Authors require contributions: Qiang Liu, Debby Dan Wang, Weiqing Guo, Yuting Huang, and Xizhao Wang. Please ensure that the full contributions of each author are acknowledged in the "Add/Edit/Remove Authors" section of our submission form.

The list of CRediT author contributions may be found here: https://journals.plos.org/ploscompbiol/s/authorship#loc-author-contributions

2) We ask that a manuscript source file is provided at Revision. Please upload your manuscript file as a .doc, .docx, .rtf or .tex. If you are providing a .tex file, please upload it under the item type u2018LaTeX Source Fileu2019 and leave your .pdf version as the item type u2018Manuscriptu2019.

3) Please upload all main figures as separate Figure files in .tif or .eps format. For more information about how to convert and format your figure files please see our guidelines:

https://journals.plos.org/ploscompbiol/s/figures

**Reviewers' comments:**

Reviewer's Responses to Questions

**Comments to the Authors:**

**Please note here if the review is uploaded as an attachment.**

Reviewer #1: This paper, “Dynamics-enhanced Molecular Property Prediction Guided by Deep Learning” by Liu et al., demonstrates a method to incorporate dynamics information into molecular property prediction. Through the construction of a frame-wise DEMR tensor to encode contacts and the injection of time information through positional encoding, the authors have shown that their dynamics-inclusive model achieves strong prediction power compared to graph-based models and language models without dynamic information.

While using stacks of transformers in this kind of task might be considered overkill and ideally warrants further justification, involving dynamics into model learning is an idea gaining appreciation in the field (especially in larger systems like protein dynamics), and I appreciate this approach significantly. In terms of quality, the authors have taken care of many details, from simulation to model training. The writing is also concise yet clear, making this a very solid work on this topic. I would like to accept this as is, but there are three minor issues to address before publishing.

1. Transferability to out-of-distribution new molecules is one of the essential tasks for these models. The authors have shown in Fig. 2 the similarities of molecules within tasks, separated into positive, negative, and inter-class. Can the authors show a similar figure comparing the training and test sets? Also, can you describe how this similarity is defined? If they are fingerprint-based, I am quite surprised that the DUD-E set similarities are this low.

2. It would be interesting to see what the learned positional encoding looks like. It would significantly enhance this paper if you can show if it looks similar to a memory kernel.

3. A presentation issue: In almost every figure (maybe except Fig. 4), the text is too small and very hard to read. Can you set your Matplotlib DPI to 300 and adjust the font sizes? Also, in the manuscript, you refer to the three sampling strategies as circled 1, 2, and 3. This circled number notation is used elsewhere (e.g., lines 303-313), which can create a minor clarity issue. You have given your three strategies very good names (Section 3.2, Lines 206-215); I suggest using these names throughout the manuscript.

Reviewer #2: The authors presented a manuscript with the title: “Dynamics-enhanced Molecular Property Prediction Guided by Deep Learning”. This manuscript presents a dynamics-enhanced molecular property prediction (MPP) framework, which couples molecular dynamics (MD) simulations with a new molecular representation named Dynamics-Enhanced Molecular Representation (DEMR). The data used in the study involves preparation of MD trajectories (10 ns per molecule) of a set of molecules taken from the Tox21 and DUD-E subsets. A frame-based atom-type/distance scheme, along with a Transformer or CNN-Transformer model is used. Impact of various sampling strategies (i.e., different temporal frequency, RMSD-based or hybrid) on model performance was studied in great details.

In my opinion, the paper addresses an important limitation in current MPP pipelines – reliance on static molecular structures – and provides publicly available datasets (~200 GB) and code. Namely, the scale of the data generation is genuinely impressive. Curating a library of 8,145 molecules with full MD trajectories is a heavy lift, and the decision to release it all on Zenodo and GitHub is a huge service to the community.

The real "hook" here is the move away from static models (it’s not exclusive as authors stated) . By pulling MD-derived conformational dynamics into MPP, the study addresses a major gap in the field exactly when people are looking for more than just 2D snapshots. The systematic deep dive into sampling – specifically comparing frequency vs. RMSD-based strategies –isn't just a technical detail; it’s a necessary roadmap for anyone trying to replicate or build on this approach.

Taking all above as the advance, I would highlight few concerns that should be addressed.

1. The novelty term is overstated (“fist application…”), MD-informed representations and ensemble-based conformer learning have appeared in binding affinity prediction and 3D-GNN literature, I believe even cited by the Authors.

The manuscript should:

- Carefully position itself relative to equivariant 3D GNNs and ensemble conformer learning.

- Clarify what is genuinely novel: the specific atom-pair-distance tensor? the systematic MD dataset? the RMSD-weighting strategy?

2. For DUD-E tasks, reported ROC-AUC values near (or exactly) 1.00 (Table 3, p. 11) are suspiciously high. DUD-E is known to allow artificial separation between actives and decoys.

The authors should clarify:

- Was scaffold split used?

- Were conformers from the same molecule ever distributed across train/test?

- Was any chemical similarity control performed?

- Without scaffold-level splitting, improvements may reflect memorization rather than dynamics learning.

3. Each molecule is simulated for 10 ns. For many flexible ligands, 10 ns may not sufficiently sample relevant conformational space. Could you discuss this and provide the explanation why 10 ns is sufficient for all molecule sizes (4–197 atoms)?

4. DEMR encodes:

- Atom-type pairs (8×8)

- Distance bins (3 ranges)

This is effectively a histogram-based 3D descriptor. Please provide explanation:

- How does this compare to modern equivariant GNNs using full 3D coordinates?

- Is the improvement due to dynamics or simply due to using 3D information?

- Is there a baseline using single static 3D conformer with the same encoding?

- What about 3D static control as the evaluation point?

5. Some of the results are reported as single averages. Given performance differences of ~0.03–0.05 ROC-AUC there are missing:

- Standard deviations across runs.

- Statistical significance testing.

- Confidence intervals.

6. Some RMSD-region weights are negative. Interpretation is unclear. Why are HLC weights sometimes negative for CNN Transformer?

7. Authors may wish to provide the discussion of sequence length sensitivity when T increases.

**********

**Have the authors made all data and (if applicable) computational code underlying the findings in their manuscript fully available?**

The PLOS Data policy requires authors to make all data and code underlying the findings described in their manuscript fully available without restriction, with rare exception (please refer to the Data Availability Statement in the manuscript PDF file). The data and code should be provided as part of the manuscript or its supporting information, or deposited to a public repository. For example, in addition to summary statistics, the data points behind means, medians and variance measures should be available. If there are restrictions on publicly sharing data or code —e.g. participant privacy or use of data from a third party—those must be specified.

Reviewer #1: Yes

Reviewer #2: Yes

**********

PLOS authors have the option to publish the peer review history of their article (what does this mean?). If published, this will include your full peer review and any attached files.

If you choose “no”, your identity will remain anonymous but your review may still be made public.

**Do you want your identity to be public for this peer review?**  For information about this choice, including consent withdrawal, please see our Privacy Policy.

Reviewer #1: No

Reviewer #2: No

[NOTE: If reviewer comments were submitted as an attachment file, they will be attached to this email and accessible via the submission site. Please log into your account, locate the manuscript record, and check for the action link "View Attachments". If this link does not appear, there are no attachment files.]

**Figure resubmission:**

While revising your submission, we strongly recommend that you use PLOS’s NAAS tool (https://ngplosjournals.pagemajik.ai/artanalysis) to test your figure files. NAAS can convert your figure files to the TIFF file type and meet basic requirements (such as print size, resolution), or provide you with a report on issues that do not meet our requirements and that NAAS cannot fix.

After uploading your figures to PLOS’s NAAS tool - https://ngplosjournals.pagemajik.ai/artanalysis, NAAS will process the files provided and display the results in the "Uploaded Files" section of the page as the processing is complete. If the uploaded figures meet our requirements (or NAAS is able to fix the files to meet our requirements), the figure will be marked as "fixed" above. If NAAS is unable to fix the files, a red "failed" label will appear above. When NAAS has confirmed that the figure files meet our requirements, please download the file via the download option, and include these NAAS processed figure files when submitting your revised manuscript.

**Reproducibility:**

To enhance the reproducibility of your results, we recommend that authors of applicable studies deposit laboratory protocols in protocols.io, where a protocol can be assigned its own identifier (DOI) such that it can be cited independently in the future. Additionally, PLOS ONE offers an option to publish peer-reviewed clinical study protocols. Read more information on sharing protocols at https://plos.org/protocols?utm_medium=editorial-email&utm_source=authorletters&utm_campaign=protocols

# Author comment: Author response to Decision Letter 1

**DOI:** 10.1371/journal.pcbi.1014515.r002

26 Jun 2026

Attachment Submitted filename: ResponseLetter.pdf

# Peer review: Decision Letter 1

**DOI:** 10.1371/journal.pcbi.1014515.r003

**Authors:** Mark Alber

30 Jun 2026

Dear Dr. Wang,

We are pleased to inform you that your manuscript 'Dynamics-enhanced Molecular Property Prediction Guided by Deep Learning' has been provisionally accepted for publication in PLOS Computational Biology.

Before your manuscript can be formally accepted you will need to complete some formatting changes, which you will receive in a follow up email. A member of our team will be in touch with a set of requests.

Please note that your manuscript will not be scheduled for publication until you have made the required changes, so a swift response is appreciated.

IMPORTANT: The editorial review process is now complete. PLOS will only permit corrections to spelling, formatting or significant scientific errors from this point onwards. Requests for major changes, or any which affect the scientific understanding of your work, will cause delays to the publication date of your manuscript.

Should you, your institution's press office or the journal office choose to press release your paper, you will automatically be opted out of early publication. We ask that you notify us now if you or your institution is planning to press release the article. All press must be co-ordinated with PLOS.

Thank you again for supporting Open Access publishing; we are looking forward to publishing your work in PLOS Computational Biology.

Best regards,

Mark Alber, Ph.D.

Section Editor

PLOS Computational Biology

Mark Alber

Section Editor

PLOS Computational Biology

***********************************************************

Reviewer's Responses to Questions

**Comments to the Authors:**

**Please note here if the review is uploaded as an attachment.**

Reviewer #1: The current revision addressed my concerns really well. I would recommend publishing this article in its current form.

Reviewer #2: Thank you for your re-submission.

**********

**Have the authors made all data and (if applicable) computational code underlying the findings in their manuscript fully available?**

The PLOS Data policy requires authors to make all data and code underlying the findings described in their manuscript fully available without restriction, with rare exception (please refer to the Data Availability Statement in the manuscript PDF file). The data and code should be provided as part of the manuscript or its supporting information, or deposited to a public repository. For example, in addition to summary statistics, the data points behind means, medians and variance measures should be available. If there are restrictions on publicly sharing data or code —e.g. participant privacy or use of data from a third party—those must be specified.

Reviewer #1: Yes

Reviewer #2: Yes

**********

PLOS authors have the option to publish the peer review history of their article (what does this mean?). If published, this will include your full peer review and any attached files.

If you choose “no”, your identity will remain anonymous but your review may still be made public.

**Do you want your identity to be public for this peer review?**  For information about this choice, including consent withdrawal, please see our Privacy Policy.

Reviewer #1: No

Reviewer #2: No

# Editor report: Acceptance letter

**DOI:** 10.1371/journal.pcbi.1014515.r004

**Authors:** Mark Alber

PCOMPBIOL-D-26-00323R1

Dynamics-enhanced Molecular Property Prediction Guided by Deep Learning

Dear Dr Wang,

I am pleased to inform you that your manuscript has been formally accepted for publication in PLOS Computational Biology. Your manuscript is now with our production department and you will be notified of the publication date in due course.

The corresponding author will soon be receiving a typeset proof for review, to ensure errors have not been introduced during production. Please review the PDF proof of your manuscript carefully, as this is the last chance to correct any errors. Please note that major changes, or those which affect the scientific understanding of the work, will likely cause delays to the publication date of your manuscript.

Soon after your final files are uploaded, unless you have opted out, the early version of your manuscript will be published online. The date of the early version will be your article's publication date. The final article will be published to the same URL, and all versions of the paper will be accessible to readers.

For Research, Software, and Methods articles, you will receive an invoice from PLOS for your publication fee after your manuscript has reached the completed accept phase. If you receive an email requesting payment before acceptance or for any other service, this may be a phishing scheme. Learn how to identify phishing emails and protect your accounts at https://explore.plos.org/phishing.

Thank you again for supporting PLOS Computational Biology and open-access publishing. We are looking forward to publishing your work!

With kind regards,

Anitha Samidurai

PLOS Computational Biology | Carlyle House, Carlyle Road, Cambridge CB4 3DN | United Kingdom ploscompbiol@plos.org | Phone +44 (0) 1223-442824 | ploscompbiol.org | @PLOSCompBiol
